# Rotenoids from the Roots of *Vicia faba* L. (Fabaceae): Structural Characterization, Cytotoxic Effects, and Molecular Docking

**DOI:** 10.1002/cbdv.202501091

**Published:** 2025-05-26

**Authors:** Victor Menezes Sipoloni, João Victor Silva‐Silva, Elthon G. Ferreira, Eric Yoshitaka Lee, João Marcos Batista Junior, Miriam Uemi, Lívia Soman de Medeiros, Paula C. Jimenez, Thiago A. M. Veiga

**Affiliations:** ^1^ Programa de Pós‐Graduação em Biologia Química Instituto de Ciências Ambientais, Químicas e Farmacêuticas, Universidade Federal de São Paulo Diadema Brazil; ^2^ Laboratório de Química Medicinal e Computacional Instituto de Física de São Carlos, Universidade de São Paulo Sao Carlos Brazil; ^3^ Departamento de Farmacologia Instituto de Ciências Biomédicas, Universidade de São Paulo Sao Paulo Brazil; ^4^ Instituto de Ciência e Tecnologia, Universidade Federal de São Paulo Sao Jose dos Campos Brazil; ^5^ Departamento de Química Instituto de Ciências Ambientais, Químicas e Farmacêuticas, Universidade Federal de São Paulo Diadema Brazil; ^6^ Departamento de Ciências do Mar Instituto do Mar, Universidade Federal de São Paulo Santos Brazil

**Keywords:** cytotoxicity, isoflavonoids, molecular docking, network pharmacology, rotenoids, *Vicia faba*

## Abstract

The chemical study of the ethanolic extract from the roots of *Vicia faba* led to the isolation of two isoflavonoids, alfalone and 8‐*O*‐methylretusine, as well as a mixture of rotenoids, including clitoriacetal and clitoriacetal B, the latter of which is reported for the first time. These compounds were characterized through nuclear magnetic resonance and vibrational circular dichroism spectroscopies, and density functional theory calculations. The rotenoid mixture exhibited cytotoxic activity against HCT‐116, MCF‐7, and 501Mel cell lines, while showing no significant toxicity to NIH/3T3 cells. The predictive analysis identified several shared therapeutic targets across colorectal cancer, breast cancer, and melanoma. Key sites, including hypoxia‐inducible factor 1‐alpha (HIF1A), estrogen receptor, heat shock protein HSP 90‐beta, and heat shock protein HSP 90‐alpha, were highlighted for their critical roles in tumor progression and therapeutic resistance. Notably, clitoriacetal demonstrated an affinity for HIF1A, suggesting its involvement in the observed antitumor effects, likely through modulation of the HIF1A pathway. These findings underscore the potential of *V. faba* root‐derived compounds as promising candidates for targeted cancer therapies.

## Graphical abstract

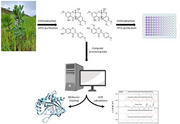

## 1 Introduction

The *Vicia* L. genus includes 130–240 annual and perennial species distributed across Europe, Asia, the Americas, and East Africa, with the Mediterranean as the first reported location [1, 2]. These plants are employed in traditional medicine to treat various diseases such as cancer, diabetes, cardiovascular diseases, diarrhea, and infertility. Besides that, they also exhibit antioxidant, antidiabetic, antiparkinsonian, and anti‐inflammatory properties [3, 4, 5].

Among *Vicia* species, *V. faba*, commonly referred to as “feijão‐fava”, “fava” or “feijão‐largo” displays an herbaceous growth pattern, reaching heights of up to 2 m [6]. Its major secondary metabolites consist of phenolic compounds, comprising approximately 85% of its composition [7]. Furthermore, it harbors alkaloids, jasmonates, organic acids, lignans, terpenoids, and flavonoids [8, 9]. A thorough investigation has unveiled the manifold benefits of flavonoids in promoting human health, including their antioxidant, antiviral, anti‐inflammatory, and anticancer effects [10, 11, 12].

Despite the identification of nearly 300 000 natural compounds through traditional methods over the past century, inherent technical constraints in natural product research have restricted their incorporation into contemporary drug discovery strategies [13]. Nonetheless, irrespective of these statistics, the ongoing pursuit of drug candidates derived from undiscovered natural products remains of utmost significance. Based on this perspective, the roots of *V. faba* L. were investigated in search of metabolites with activity against human tumor cell lines.

**FIGURE 1:**
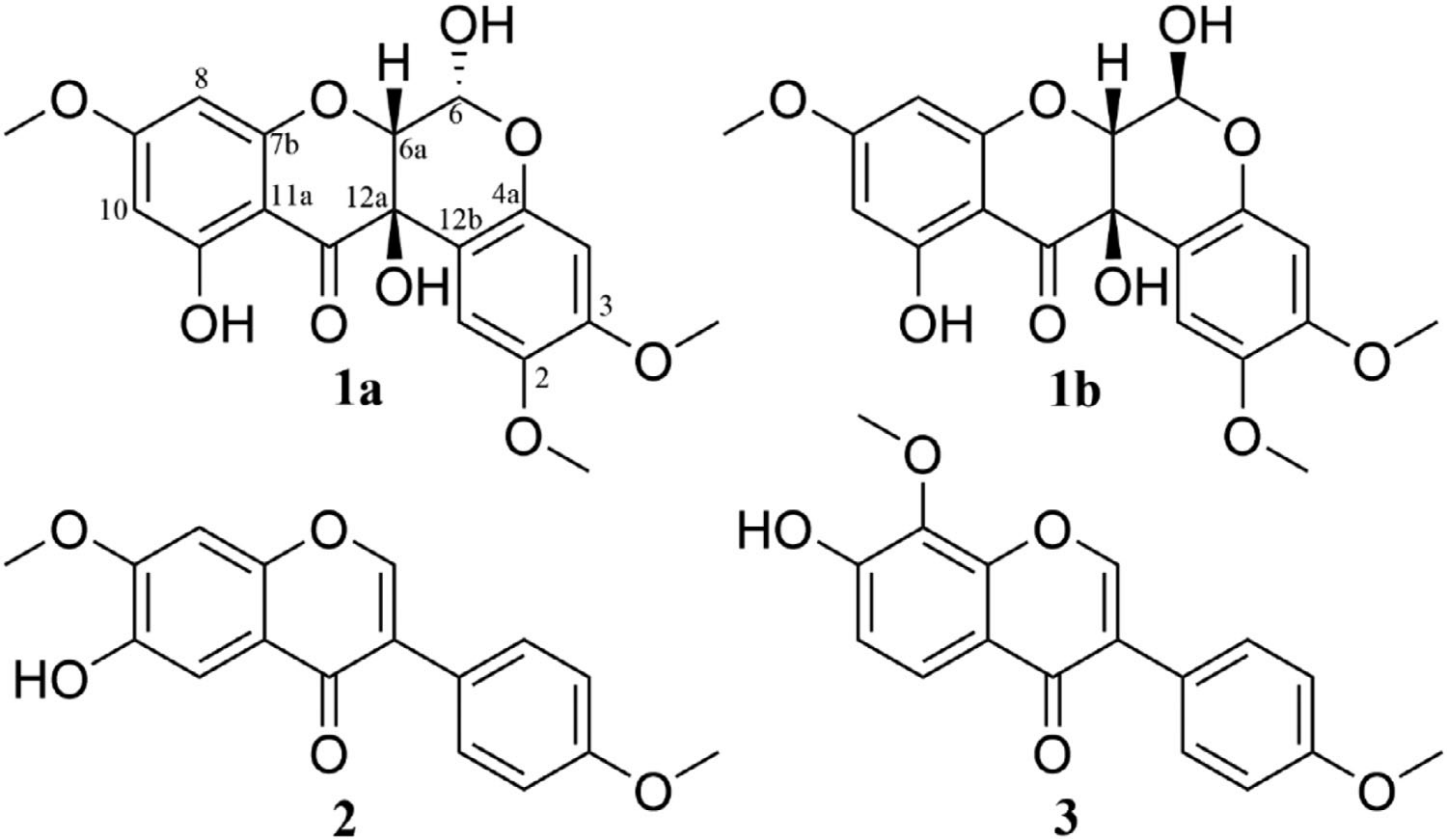
Isolated and characterized metabolites from the roots of *V. faba* L.

By using chromatographic and spectroscopic methods, we successfully isolated and characterized a pair of diastereoisomeric rotenoids (**1a** and **1b**), along with two known isoflavones (**2** and **3**) (Figure 1). These compounds, which were fully characterized by comprehensive nuclear magnetic resonance (NMR) spectroscopy and vibrational circular dichroism (VCD) data, are the first isoflavonoids and rotenoids reported within the *Vicia* genus. Following isolation, all compounds were evaluated for cytotoxicity against a panel of tumor and non‐tumor cell lines, as part of our efforts to identify potential new anticancer candidates. Besides that, prediction studies were performed for the rotenoids to characterize their therapeutic targets across colorectal cancer, breast cancer, and melanoma.

## 2 Results and Discussion

### 2.1 Compounds Isolation and Structures Elucidation

The dried and powdered roots (1874.0 g) of V. faba were extracted with absolute ethanol over nine days, with the solvent being changed every three days, to obtain the ethanolic extract (90.0 g). Liquid‐liquid fractionation procedures yielded the dichloromethane fraction (FDR, 3.9 g), which was evaluated against HCT‐116 (human colorectal carcinoma) cell lines, presenting an IC_50_ of 16.73 µg/mL (95% confidence interval [CI] 10.18–28.53 µg/mL; R^2^ 0.8928). To isolate cytotoxic metabolites, we further purified FDR through column chromatography (CC) and preparative high‐performance liquid chromatography (HPLC), leading to the characterization of four compounds: **1a** and **1b**, a mixture of diastereoisomers with a rotenoid core, which was identified as clitoriacetal [14] and clitoriacetal B (first described here), respectively, along with two isoflavones, alfalone (**2**) [15] and 8‐O‐methylretusin (**3**) [16].

The pair of rotenoids was isolated as a yellow amorphous solid. Its molecular formula (C_19_H_18_O_9_) was determined by analysis of the (+)‐high‐resolution electrospray ionization mass spectrometry (Figure S1) peak at [M+H]^+^ *m/z* 391.1021. The 1H NMR spectrum (CDCl_3_, 500 MHz, Figure S2) revealed the presence of two compounds, likely a pair of isomers, as indicated by duplicated signals with varying intensities. All signals were successfully identified and assigned, with the major isomer designated as **1a** and the minor one as **1b**.

**FIGURE 2:**
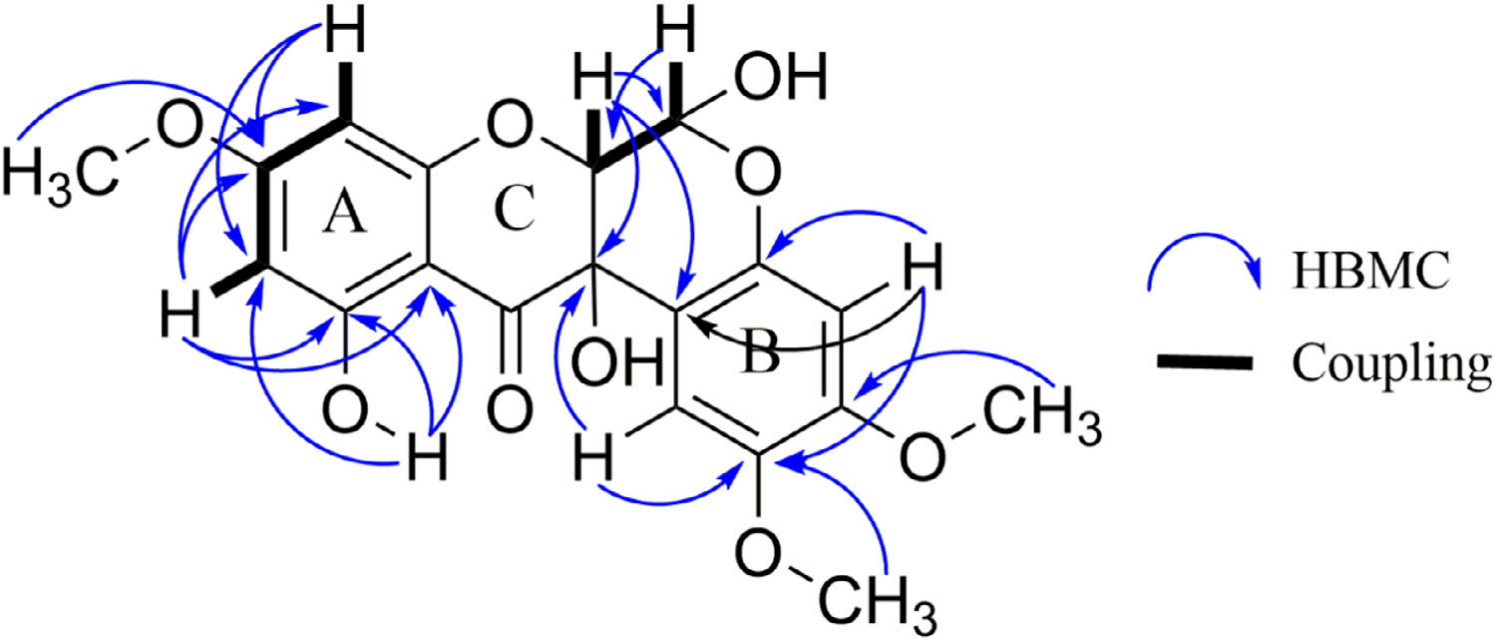
Heteronuclear multiple bond correlation (HMBC) correlations for compounds **1a** and **1b** and vicinal coupling in six‐membered rings on rings B and D.

Upon examination of the ^1^H NMR spectrum (Figure S2), a distinctive signal was observed at *δ* 11.44, corresponding to the chelated hydroxyl at position C11 for **1a**, and at *δ* 11.39 for **1b**. Through the heteronuclear multiple bond correlation (HMBC) experiment (Figure S3) for **1a**, spatial correlation of the 11‐OH signal with carbons at *δ* 164.47, *δ* 100.0, and *δ* 95.94 was observed (Figure 2 and Table 1), attributed to C11, C11a, and C10 of **1a**, respectively. For **1b**, similar correlations were observed, with corresponding carbons at *δ* 164.44 (C11), *δ* 99.85 (C11a), and *δ* 95.99 (C10). Additionally, signals in the region of the aromatic nuclei were observed: two doublets integrating for ^1^H each at *δ* 6.00 and 6.084 for **1a**, and signals at *δ* 5.97 and 6.082 for **1b**, both with *J* = 2.0 Hz, indicating a meta coupling. These signals were attributed to the H10 and H8 protons observed in ring A, respectively.

**TABLE 1:** **^1^** H‐NMR (500 MHz, CDCl_3_) and ^13^C‐NMR (125 MHz, CDCl_3_) spectroscopic data for rotenoids **1a** and **1b**.

	1a				1b			
	*δ*_C_, type	*δ*_H_ (*J* in Hz)	HMBC^a^	2D NOESY^b^	*δ*_C_, type	*δ*_H_ (*J* in Hz)	HMBC^a^	2D NOESY^b^
**1**	108.56	6.65 (s)	69.71 (C12a); 144.49 (C2)	3.75 (2‐OMe)	108.76	6.68 (s)	68.03 (C12a); 144.54 (C2); 151.91 (C3)	3.76 (2‐OMe)
**2**	144.49	—	—	—	144.54	—	—	—
**3**	151.55	—	—	—	151.91	—	—	—
**4**	101.16	6.52 (s)	144.49 (C2); 107.71 (C12b); 148.11 (C4)	3.82 (3‐OMe)	102.00	6.56 (s)	144.54 (C2); 107.38 (C12b); 148.13 (C4a)	3.83 (3‐OMe)
**4a**	148.11	—	—	—	148.13	—	—	—
**6**	90.4	5.62 (d, 2.0 Hz)	77.06 (C6)	4.56 (H6a); 3.82 (3‐OMe)	91.58	5.73 (d, 1.0 Hz)	74.63 (C6)	4.71 (H6a); 3.83 (3‐OMe)
**6a**	77.06	4.56 (d, 2.0 Hz)	69.71 (C12a); 107.71 (C12b); 194.19 (C12)	5.62 (H6)	74.63	4.71 (d, 1.0 Hz)	68.03 (C12a); 107.38 (C12b); 193.15 (C12)	5.73 (H6)
**7b**	160.54	—	—	—	161.01	—	—	—
**8**	94.81	6.00 (d, 2.0 Hz)	169.32 (C9); 95.94 (C10)	3.786 (9‐OMe)	94.75	5.97 (d, 2.0 Hz)	169.21 (C9); 95.91 (C10)	3.785 (9‐OMe)
**9**	169.32	—	—	—	169.21	—	—	—
**10**	95.94	6.084 (d, 2.0 Hz)	94.81 (C8); 169.32 (C9); 164.47 (C11); 100.0 (C11a)	3.786 (9‐OMe)	95.91	6.082 (d, 2.0 Hz)	94.75 (C8); 169.21 (C9); 164.44 (C11); 99.85 (C11a)	3.785 (9‐OMe)
**11**	164.47	—	—	—	164.44	—	—	—
**11a**	100.00	—	—	—	99.85	—	—	—
**12**	194.19	—	—	—	193.15	—	—	—
**12a**	69.71	—	—	—	68.03	—	—	—
**12b**	107.71	—	—	—	107.38	—	—	—
**2‐OMe**	56.38	3.75 (s)	—	6.65 (H1)	56.36	3.76 (s)	—	6.68 (H1)
**3‐OMe**	55.99	3.82 (s)	—	6.52 (H4); 5.62 (H6)	55.97	3.83 (s)	—	6.56 (H4)
**9‐OMe**	55.93	3.786 (s)	169.32 (C9)	6.00 (H8); 6.084 (H10)	55.93	3.785 (s)	169.21 (C9)	5.97 (H8); 6.082 (H10)
**11‐OH**	—	11.44 (s)	95.94 (C10); 164.47 (C11); 100.0 (C11a)	—	—	11.39 (s)	95.91 (C10); 164.44 (C11); 99.85 (C11a)	—
**6‐OH**	—	—	—	—	—	4.95 (sl)	—	—
**12‐OH**	—	—	—	—	—	—	—	—

^a^ HMBC correlations, optimized for 10 Hz, are from proton(s) stated to the indicated carbon.

^b^ 2D NOESY correlations, optimized for 400 ms, are from proton(s) stated to the indicated proton.

The heteronuclear single quantum coherence (HSQC) experiment (Figure S4) revealed couplings between H8 and the carbon at *δ* 94.81, as well as between H10 and *δ* 95.94 for **1a**. Similarly, for **1b**, the same pattern was observed: H8 (*δ* 5.97) and H10 (*δ* 6.082) with *δ* 94.75 and *δ* 95.91, respectively. In the HMBC experiment (Figure S3), H10 exhibited *^2^J* correlations with carbons at *δ* 164.47 and *δ* 169.32 for **1a**, corresponding to the oxidized carbons C11 and C9, respectively. Additionally, *^3^J* correlations were observed with carbons at *δ* 100.0 (C11a) and *δ* 94.81 (C8). For **1b**, similar correlations were observed with carbons at *δ* 99.85 and *δ* 94.75.

In both compounds, H8 showed a *^2^J* correlation with the signal of C9 and a *^3^J* correlation with the signals of C10 and C11a. A singlet in the methoxy region at *δ* 3.786 for **1a** displayed an HMBC correlation with the carbon signal at *δ* 169.32 (C9), while the signal at *δ* 3.785 for **1b** correlated with the carbon at *δ* 169.21 (C9). These observations suggested that the A ring of the isomers exhibits a 1,2,3,5‐tetrasubstituted pattern.

For ring B, an examination of the correlation spectroscopy experiment (Figure S5) revealed no coupling in the aromatic region. Consequently, the signals at *δ* 6.65 and *δ* 6.52, each integrating to 1H (Figure S2), were assigned to **1a**, while the signals at *δ* 6.68 and *δ* 6.56 were attributed to **1b**. These singlets suggested a para‐type substitution pattern. The HSQC experiment (Figure S4) revealed direct correlations of these protons with the carbons at *δ* 108.52 and *δ* 101.16 for **1a**, and at *δ* 108.78 and *δ* 102.00 for **1b**, respectively.

The remaining singlets in the region of the methoxy groups at *δ* 3.82, *δ* 3.75 for **1a**, and *δ* 3.76, *δ* 3.83 for **1b**, were assigned to positions, C2 and C3, respectively. The signals at *δ* 3.83 and *δ* 3.76 showed correlations, via HMBC (Figure S3), with the carbons at *δ* 144.54 (C2) and *δ* 151.91 (C3), for **1b**. To respect the para pattern and the correlations observed in the HMBC contour plot, the signal at *δ* 6.52 was assigned to H4 in compound **1a**. This assignment was based on correlations with C3 at *δ* 151.55 and with C12b at *δ* 107.71. For compound **1b**, the signal at *δ* 6.56 was assigned to H4 due to its correlations with C4a (*δ* 148.13) and with C2 (*δ* 144.54) and C12b (*δ* 107.38). The signal at *δ* 6.68 was attributed to H1 in **1b**, as it showed a correlation with C2, C3, and C12a. In **1a**, the signal at *δ* 6.65 was assigned to H1 because of its *^2^J* correlation with C2 and C12a. These observations suggested that ring B has a 1,2,4,5‐tetrasubstituted pattern (Figure 2).

In the region corresponding to hydrogens attached to oxidized sp^3^ carbons, four doublets were observed (Figure S2): *δ* 4.56 and *δ* 5.62 with *J* = 2 Hz for **1a**, and at *δ* 4.71 and *δ* 5.73 with *J* = 1 Hz for **1b**. These *J*‐values are characteristic of vicinal coupling in six‐membered rings (Figure 2), this difference in the coupling constant indicates the presence of epimers at position C6. The relatively shifted signals at *δ* 5.62 and *δ* 5.73 suggest the presence of a doubly oxidized methine group, indicative of a hemiacetal moiety, likely due to the presence of a hydroxy group at C6.

HSQC experiment (Figure S4) revealed that H6a (*δ* 4.56) is attached to the carbon at *δ* 77.06 and *δ* 5.62 (H6) to the carbon at *δ* 90.4 for **1a**. For **1b**, H6a (*δ* 4.71) is connected to the carbon at *δ* 74.63 and *δ* 5.73 (H6) to the carbon at *δ* 91.58. To verify these positions, the HMBC (Figure S3) experiment revealed a *^2^J* correlation for H6 (**1a**) with C6a (*δ* 77.06). In its turn, H6a (*δ* 4.56) correlated with C1a (*δ* 107.71) and C12 (*δ* 194.19). Similarly, for **1b**, H6a showed correlations with C12b (*δ* 107.38) and C12 (*δ* 193.15).

**FIGURE 3:**
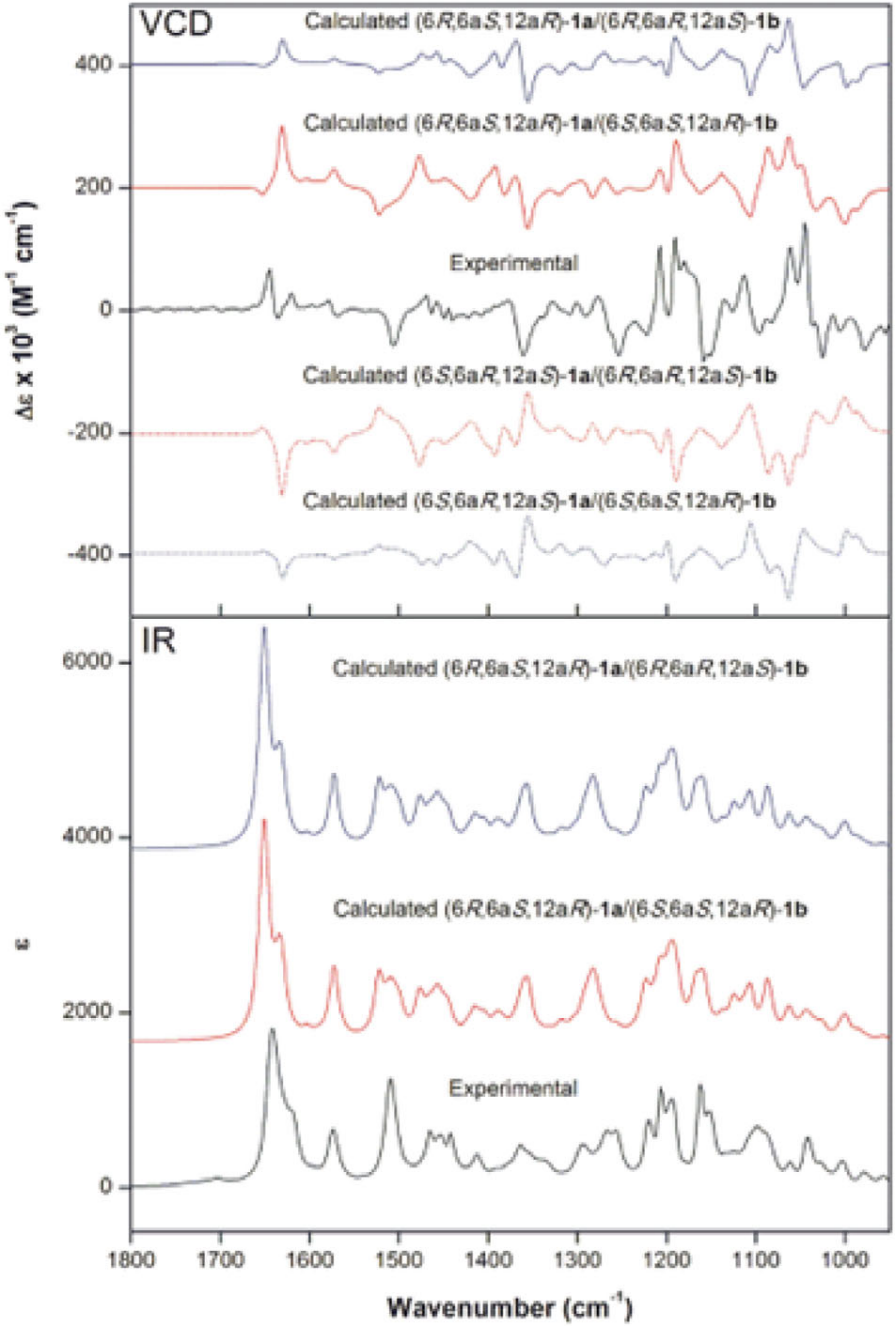
Comparison of experimental infrared (IR) and vibrational circular dichroism (VCD) spectra (black trace) for the mixture **1a**/**1b** with calculated [B3PW91/PCM(CHCl_3_)/6‐311G(d,p)] data (blue and red traces) for different combinations of a 2:1 mixture of **1a** and **1b**. Calculated IR spectra omitted for enantiomers. Spectra offset for clarity.

Nuclear Overhauser enhancement spectroscopy two‐dimensional (2D) experiments were also performed to confirm the long‐distance correlations (Figures S6 and S7). Data showed that, for both isomers **1a** and **1b**, the nuclei in 9‐OMe correlated with those at positions H8 and H10, confirming the assignment of ring A. For ring B, correlations were observed between the nucleus at H4 and 3‐OCH_3_, as well as between H1 and 2‐OCH_3_, supporting the correct assignment of the methoxy groups. In ring D, H6 presented spatial correlation with the methoxy group at 3‐OMe, and also with H6a. Following the assignment of the relative configuration of both **1a** and **1b**, VCD spectroscopy and density functional theory (DFT) calculations were used to establish their absolute configurations [17]. Given the evidence about the epimeric nature of **1a** and **1b**, calculations were initially performed for the arbitrarily chosen configurations (6R,6aS,12aR) for **1a** and (6S,6aS,12aR) for **1b**. As the experimental VCD spectrum represents a 2:1 mixture of **1a** and **1b**, the calculated infrared (IR) and VCD spectra were weighted accordingly considering all different combinations of the configurations of **1a** and **1b**. The results clearly favored the (6aS,12aR) configuration (Figure 3). Nevertheless, 2:1 combinations of (6R,6aS,12aR)‐1a and (6S,6aS,12aR)‐1b, as well as (6R,6aS,12aR)‐1a and (6R,6aR,12aS)‐1b, resulted in nearly superimposable spectra due to the intensity dominance of the major compound **1a**, preventing the unambiguous assignment of **1b** (Figure 3). Comparisons of the calculated IR and VCD spectra of individual compounds with experimental data for the mixture, however, demonstrated good agreements for both (6R,6aS,12aR)‐1a and (6S,6aS,12aR)‐1b (Figures S8 and S9). Therefore, these configurations were assigned to the epimers **1a** and **1b**, respectively.

### 2.2 Cytotoxic Effects of the Isolated Compounds

The mixture of **1a** and **1b** was evaluated for in vitro cytotoxic activity against the human tumor cell lines HCT‐116, MCF‐7, and 501Mel, and against the non‐tumor murine fibroblast NIH/3T3. The results, presented in Table 2, demonstrated that the isomers exhibited greater cytotoxicity against MCF‐7 cells, with an IC_50_ of 3.73 µM. In contrast, the **1a**/**1b** showed an IC_50_ greater than 50.00 µM against the non‐tumorigenic NIH/3T3 cells, yielding a selectivity index (SI) > 13 for the aforementioned tumor cell line. For HCT‐116 and 501Mel cells, IC_50_ values were observed at 7.10 µM and 15.21 µM, corresponding to SI of >7 and >3, respectively. Compounds **2** and **3** showed no cytotoxic activity in these experiments.

**TABLE 2:** Cytotoxic effects of the mixture **1a** and **1b**.

Compound	Parameters	Cell lines			
		HCT‐116	MCF‐7	501MeI	NIH/3T3
**1a**/**1b**	**IC_50_ (µM)**	7.10	3.73	15.21	> 50.00
	**CI95% (µM)**	3.97–13.53	1.38–14.91	5.88–65.92	—
	**R^2^**	0.8943	0.8062	0.7159	—
	**SI** ^a^	>7.04	>13.40	>3.29	—
**Doxorubicin**	**IC_50_ (µM)**	0.05	0.03	0.12	0.35
	**CI95% (µM)**	0.03–0.09	0.01–0.05	0.06–0.22	0.05–2.83
	**R^2^**	0.9789	0.9700	0.9819	0.8600
	**SI** ^a^	7.0	11.67	2.91	—

^a^ Selectivity Index (SI) = IC_50 respective cell line_/ IC_50 NIH/3T3 cell line._

When comparing the results of the mixture **1a** and **1b** with doxorubicin, a reference chemotherapy drug, it is observed that doxorubicin exhibited potent cytotoxicity against the tumor cell lines HCT‐116, MCF‐7, and 501Mel. The absence of significant cytotoxicity observed in NIH/3T3 cells (IC_50_ > 50.00 µM) is a notable finding, indicating that the mixture **1a** and **1b** may exert a selective action against tumor cells, with less impact on non‐tumor cells. These results suggest that the compounds have a pronounced preference for tumor cells, which reduces the risk of adverse effects on healthy tissues. This characteristic is particularly advantageous in the development of anticancer therapies, where it is crucial to achieve high efficacy against cancer cells while minimizing toxicity to healthy cells.

The lack of activity observed for compounds **2** and **3** in these assays suggests that the structure and specific functional groups present in compounds **1a** and **1b** are essential for their cytotoxic effects. These structural differences likely influence the ability of **1a** and **1b** to bind to specific cellular targets, emphasizing the importance of the rotenoid core and associated functional groups in mediating their potent cytotoxic properties. Moreover, interactions between different compounds can enhance therapeutic effects and produce synergistic outcomes [18]. In this context, combining **1a** and **1b** may further potentiate their cytotoxicity, with their distinct functional groups complementing each other to amplify their overall effects. This synergy could explain the potent anticancer activity observed in the mixture, reinforcing the idea that the combined action of compounds in an extract is often more effective than the individual effects of each component [19].

An additional hypothesis to explain the antitumor activity of **1a** and **1b** is that isomer **1a**, due to its higher concentration in the mixture, may be the primary responsible for the cytotoxic effects [20]. To investigate this possibility, in silico studies were performed, focusing on molecular docking and their cancer‐related molecular targets, in order to better understand the contribution of both rotenoids to the inhibitory response observed in the in vitro study.

### 2.3 Screening of Common Genes for Rotenoids and Colorectal Cancer, Breast Cancer, and Melanoma

In this study, multiple databases and computational approaches were employed to identify genes associated with cancer pathogenesis and their respective targets, with an emphasis on predicting the affinity of **1a** and **1b**. Target prediction for the rotenoids was conducted using a combination of tools, including SwissTargetPrediction, SEA, Way2Drug PASS Online, TargetNet, and SuperPred. Thus, 231 targets were identified. A comprehensive search using the keywords “colorectal cancer”, “breast cancer” and “melanoma” in databases such as the OMIM, GeneCards, DrugBank, and Therapeutic Target Database resulted in a total of 8340 target genes to colorectal cancer, 14338 to breast cancer, and 8189 to melanoma. Analysis of the target genes associated with **1a** and **1b**, as well as cancer‐related targets, identified an overlap of 152 genes, as depicted in Figure S10.

### 2.4 Protein–Protein Interaction Network Analysis

To confirm a direct correlation among the targets, these 152 intersection targets were introduced into the STRING database to obtain a protein–protein interaction (PPI) network diagram (Figure S11A). The PPI network data was imported into Cytoscape and analyzed using the cytoHubba plugin with the Maximal Clique Centrality (MCC) ranking method. This analysis identified the top 20 targets as key in cancer treatment with **1a** and **1b**, including hypoxia‐inducible factor 1‐alpha (HIF1A), estrogen receptor (ESR1), heat shock protein HSP 90‐beta (HSP90AB1), and heat shock protein HSP 90‐alpha (HSP90AA1) (Figure S11B).

HIF1A, a key regulator of the hypoxic response, promotes angiogenesis and metabolic adaptation [21], promotes epithelial‐mesenchymal transition and tumor survival [22], and therapeutic resistance [23]. It also regulates genes such as interleukin (IL)‐6, IL‐8, and MDR1, which impact chemotherapy efficacy [24].

ESR1 encodes estrogen receptor‐α (ERα), a crucial regulator of cell proliferation in ER+ breast cancer through estrogen‐dependent signaling pathways [25]. However, mutations in ESR1 can lead to ligand‐independent activation of ER, a key mechanism driving resistance to aromatase inhibitors in ER‐positive breast cancer [26]. This resistance is further exacerbated by the cross‐talk between receptor tyrosine kinases (RTKs) and the PI3K‐AKT‐mTOR pathway, which impairs the effectiveness of treatment [27]. Consequently, ESR1 has become a critical target for investigating the underlying resistance mechanisms and for the development of more effective therapies [28].

HSP90AB1 plays a critical role in oncogene activation and tumor cell survival by stabilizing mutated proteins and preventing their degradation [29]. Its overexpression not only supports tumor cell survival but also contributes to metastasis and resistance to therapy by inhibiting apoptotic pathways and the DNA damage response, potentially through the regulation of kinases [30]. This makes HSP90AB1 a promising target for therapeutic intervention, with inhibition offering a strategy to enhance treatment outcomes [31]. Similarly, HSP90AA1 contributes to tumor progression by stabilizing proteins vital for cell survival [32]. It plays an important role in resistance to apoptosis and therapeutic resistance, as well as in regulating cell differentiation and signaling pathways [33].

Together, these targets highlight the strategic importance of HIF1A, ESR1, HSP90AB1, and HSP90AA1 in cancer treatment. Their involvement in therapy resistance makes them critical targets for enhancing therapeutic strategies, including those utilizing phytochemicals [34, 35, 36, 37].

### 2.5 Gene Ontology Enrichment and Kyoto Encyclopedia of Genes and Genomes Pathway Analysis

**FIGURE 4:**
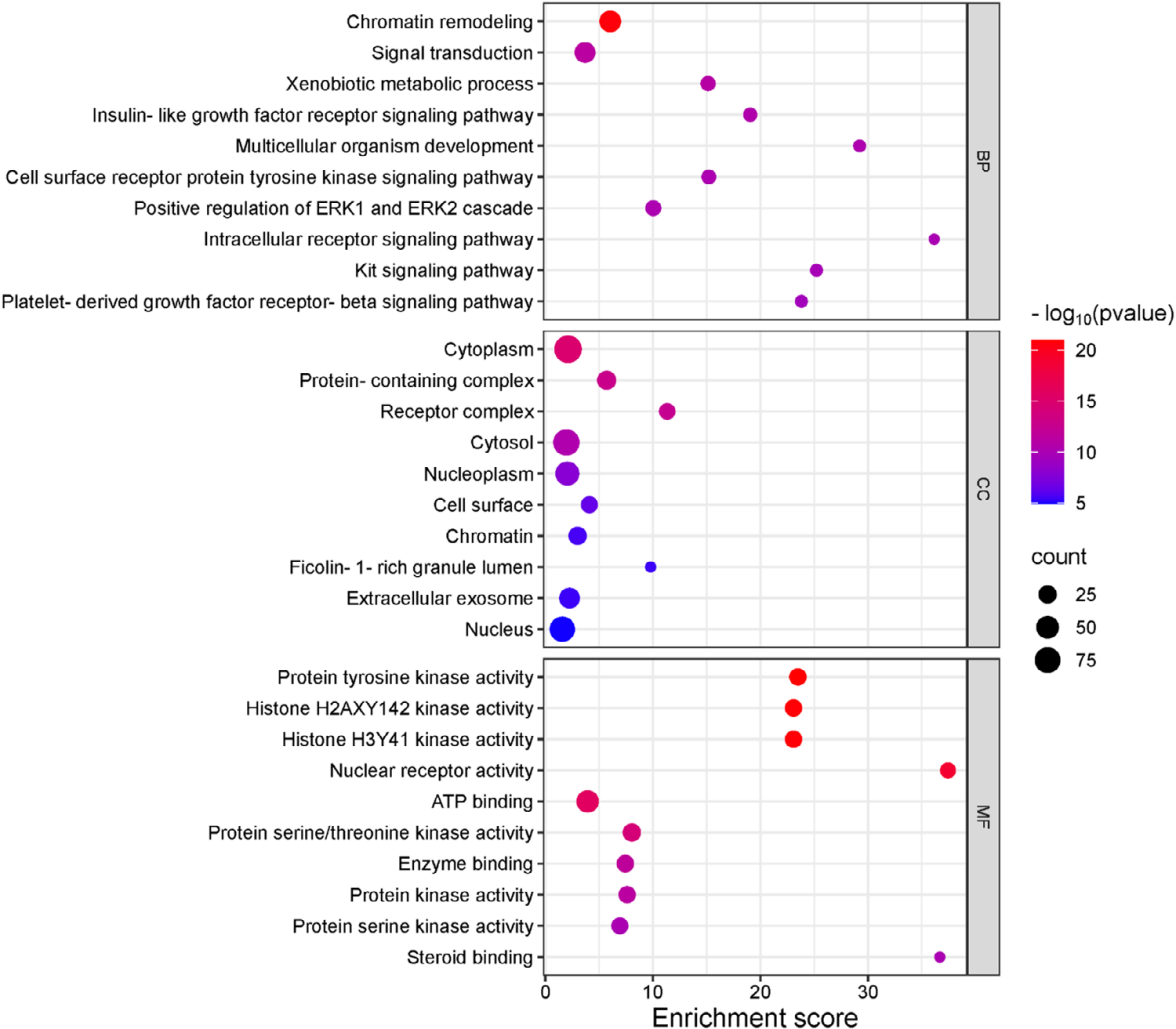
Gene ontology (GO) enrichment analysis. Bubble plots of the top 10 significant biological processes (BP), cellular components (CC), and molecular functions (MF) of the mixture of **1a** and **1b** in cancer treatment.

**FIGURE 5:**
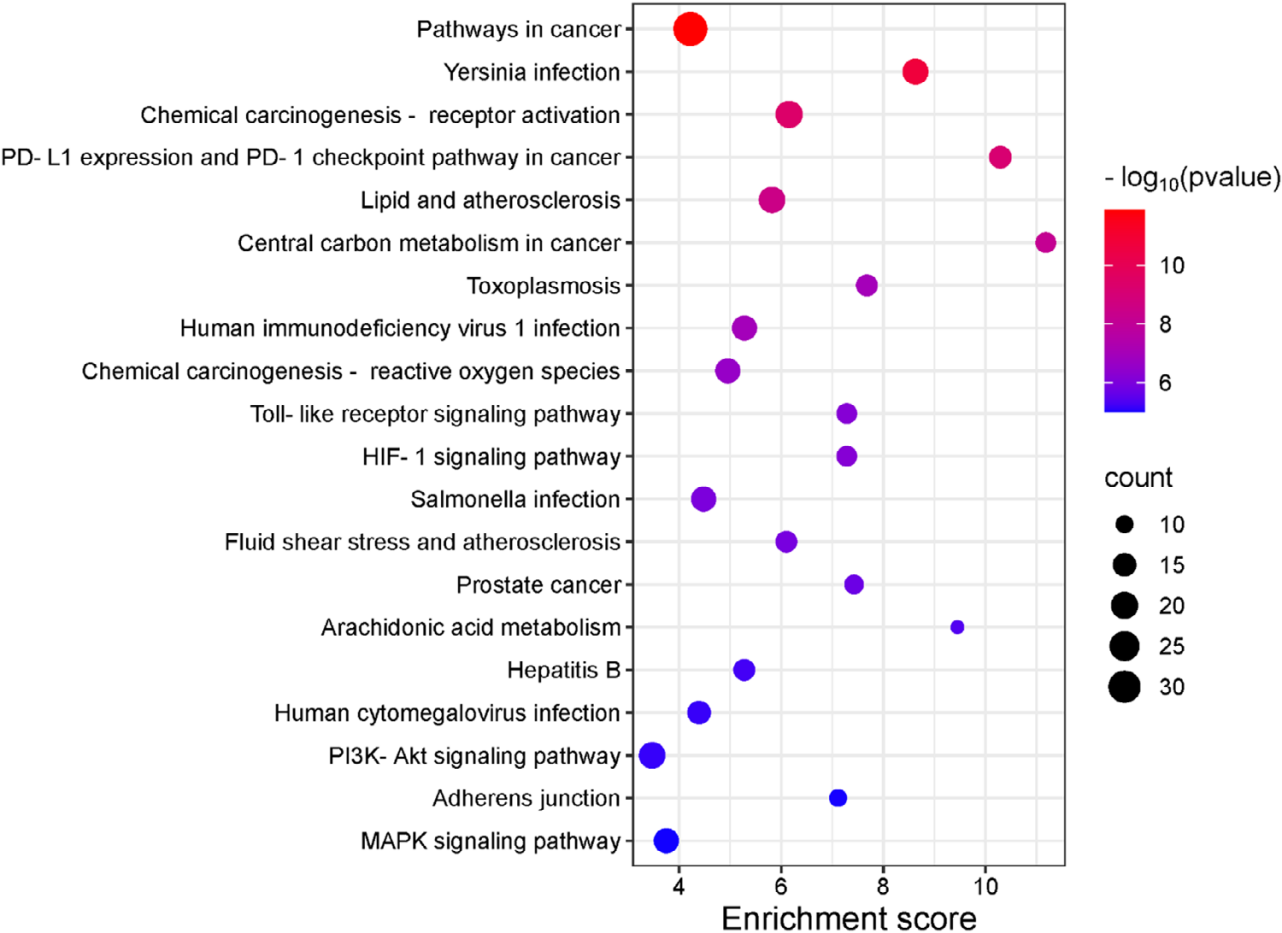
Kyoto Encyclopedia of Genes and Genomes (KEGG) enrichment analysis. Bubble plots of the top 20 significant signaling pathways of the mixture of **1a** and **1b** in cancer treatment.

To investigate the potential functional mechanisms of the 152 intersecting targets, gene ontology (GO) enrichment analysis was performed to classify biological processes (BP), cellular components (CC), and molecular functions (MF), along with the Kyoto Encyclopedia of Genes and Genomes (KEGG) pathway analysis using the DAVID database. Significant enrichment was observed for 306 of 432 BPs, 40 of 64 CCs, and 156 of 191 MFs (*p* < 0.05), with the GO analysis highlighting the top 10 enriched terms in each category using a bubble plot (Figure 4). BP terms included the chromatin remodeling, signal transduction, and xenobiotic metabolic process, while CC terms featured the cytoplasm, protein‐containing complex, and receptor complex, and MF terms involved protein tyrosine kinase activity, histone H2AXY142 kinase activity, and histone H3Y41 kinase activity. KEGG pathway analysis identified 122 pathways, 107 of which were statistically significant (*p* < 0.05). The target genes were further analyzed, focusing on the top 20 associated pathways (Figure 5 and Table S2). Among these, notable pathways included pathways in cancer, yersinia infection, and chemical carcinogenesis—receptor activation, which may serve as key interaction pathways contributing to their combined anticancer effects.

The GO and KEGG enrichment analysis provided insights into the functional mechanisms of the 152 identified targets. Among the biological processes, chromatin remodeling, signal transduction, and xenobiotic metabolism stood out, as they regulate gene expression and cellular communication. Chromatin remodeling is often altered in tumor cells [38], while signal transduction, modulated by metabolite sensing, can influence tumor progression [39]. Additionally, alterations in xenobiotic metabolism are associated with drug resistance and carcinogenesis [40].

The presence of targets in the cytoplasm and in protein complexes suggests that these targets regulate cellular adaptation and the dynamics of proteins essential for cellular function and tumor response [41]. Additionally, the presence of targets in receptor complexes indicates that these targets may activate critical signaling pathways, such as tyrosine kinase pathways, involved in the proliferation and survival of tumor cells [42]. Molecular functions, such as histone modifications [43] and tyrosine kinase activity [44], play a crucial role in regulating the cell cycle and responding to DNA damage, processes that influence cancer cell survival.

The KEGG pathway analysis identified pathways in cancer [45] and chemical carcinogenesis receptor activation as key mechanisms regulating tumor growth and metastasis. Chemical carcinogenesis receptor activation involves the metabolic activation of carcinogens [46], leading to DNA damage and mutations that drive tumor initiation and progression. The interaction of targets with these pathways can influence cellular responses to exogenous agents, including anticancer treatments.

The PPI network analysis suggests that the targets may form regulatory complexes in critical signaling pathways, impacting tumor progression. The combination of GO, KEGG, and PPI results highlights the targets as key components in molecular networks that control essential cellular functions in cancer, providing a foundation for the development of targeted therapies.

### 2.6 Molecular Docking Simulation and Validation

Molecular docking simulations were performed for four selected targets based on the PPI network analysis, which identified HIF1A, ESR1, HSP90AB1, and HSP90AA1 as key proteins involved in cancer treatment with **1a** and **1b**. These targets were prioritized due to their central roles in the network and their relevance in cancer biology, *as* determined by cytoHubba's MCC ranking method. To enable detailed interaction studies, the PDB structures selected for docking were HIF1A (PDB ID 6YW3, 2.28 Å), ESR1 (PDB ID 2IOG, 1.60 Å), HSP90AB1 (PDB ID 5UCJ, 1.69 Å), and HSP90AA1 (PDB ID 3WHA, 1.30 Å), chosen for their high resolution and the availability of co‐crystallized ligands, which are crucial for accurate analysis of potential binding interactions.

The rotenoids demonstrated exclusive affinity for HIF1A (PDB ID 6YW3), with no significant interactions with other targets, suggesting a high specificity for this protein. Among the evaluated compounds, **1a** showed a binding affinity for the active site of 6YW3, supporting the hypothesis of a stable interaction between **1a** and HIF1A, and justifying its selection for further analysis, as shown in Table 3.

**TABLE 3:** Docking simulation for the mixture of **1a** and **1b** and targets of cancer.

Target	PDB ID	RMSD	Compounds	Docking Score
HIF1A	6YW3	0.1186	1a	57.72
			1b	11.56
			OGA^a^	55.53
ESR1	2IOG	0.3579	1a	53.29
			1b	46.69
			IOG^a^	128.87
HSP90AB1	5UCJ	0.1879	1a	49.14
			1b	50.67
			KU3^a^	64.63
HSP90AA1	3WHA	0.5362	1a	54.43
			1b	56.05
			WHA^a^	77.52

^a^ co‐crystallized ligand; **1a**: Clitoriacetal; **1b**: Clitoriacetal B; **OGA**: N‐Oxalylglycine; **IOG**: N‐[(1r)‐3‐(4‐Hydroxyphenyl)‐1‐Methylpropyl]‐2‐[2‐Phenyl‐6‐(2‐Piperidin‐1‐Ylethoxy)‐1h‐Indol‐3‐Yl]acetamide; **KU3**: (5‐Fluoroisoindolin‐2‐yl)(4‐hydroxy‐5‐isopropylbenzo[d]isoxazol‐7‐yl)methanone; **WHA**: 4‐{[4‐amino‐6‐(5‐chloro‐1H,3H‐benzo[de]isochromen‐6‐yl)‐1,3,5‐triazin‐2‐yl]sulfanyl}butanamide. GoldScore with rescoring by ChemScore was chosen as a function to evaluate binding efficiency.

Biologically, HIF1A is a master transcriptional regulator of cellular responses to hypoxia, a hallmark of the tumor microenvironment [47]. Its activation promotes processes critical to tumor progression, including angiogenesis (via VEGF upregulation), metabolic reprogramming through enhanced glycolysis and suppression of mitochondrial activity, cell survival, and resistance to chemotherapy and radiotherapy [48]. Importantly, sustained HIF1A activity has been associated with tumor aggressiveness and poor prognosis in various cancers, including breast, lung (NSCLC), colorectal, pancreatic, prostate, and gastric carcinomas, as well as gliomas, melanomas, and ovarian cancers [49]. Therefore, inhibiting HIF1A can compromise the adaptive responses that allow cancer cells to thrive in low‐oxygen (hypoxic) environments, which may lead to reduced tumor growth, decreased invasiveness, and enhanced resistance to treatment [50]. In this context, HIF1A is not only a relevant marker of tumor biology but also a promising therapeutic target in oncology.

**FIGURE 6:**
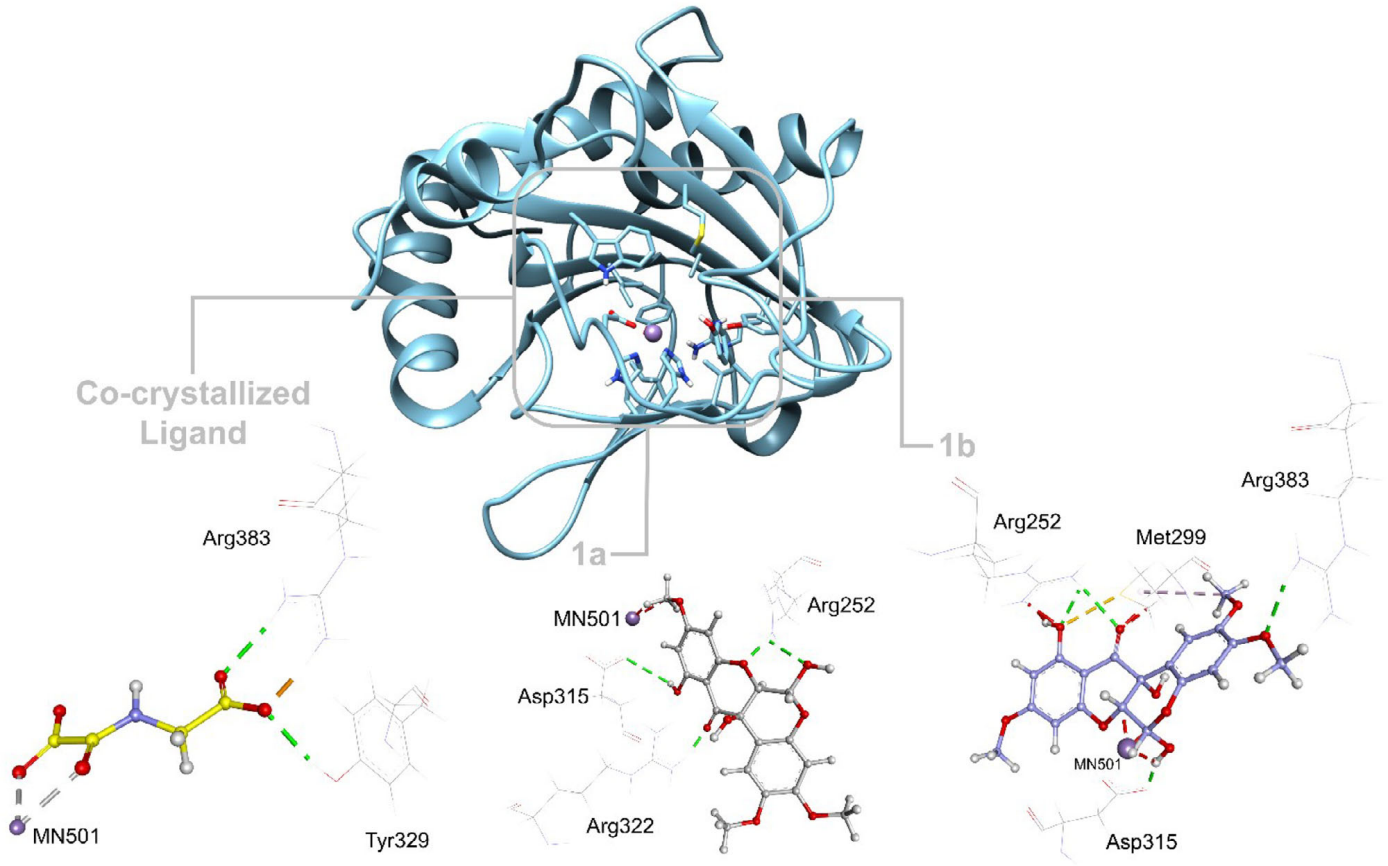
Analysis of the interaction of the mixture of **1a** and **1b** with the active site of the 6YW3 protein target. The figure was generated using UCSF Chimera v1.18 and Discovery Studio Visualizer v24.1.0.23298 (BIOVIA).

The interactions and binding residues of the predicted protein‐ligand complexes for isomers **1a** and **1b** are outlined in Table 4 and Figure 6, highlighting key molecular interactions that stabilize binding. **1a** forms hydrogen bonds with ARG252, ASP315, and ARG322, and engages in hydrophobic interactions with residues like VAL241, TYR310, and HIS413, suggesting a favorable binding mode within the active site, similar to the co‐crystallized ligand (OGA). These interactions indicate a stable interaction with the active site of 6YW3, which may modulate the HIF1A signaling pathway, crucial in cancer and cellular adaptation to hypoxic stress [51]. In contrast, **1b** exhibited weaker affinity and fewer significant interactions with 6YW3, suggesting it may have a lesser role in the observed biological activity. The data support that **1a** is likely the main contributor to the in vitro antitumor effects, possibly through inhibition of the HIF1A signaling pathway, rather than a synergistic effect between the compounds.

**TABLE 4:** Docking score and interactions of the mixture of **1a** and **1b** after docking at the hypoxia‐inducible factor 1‐alpha (HIF1A) (PDB ID 6YW3).

Compounds	Docking Score	Bond Category	Residues in Contact	Interaction Types	Distance (Å)
**1a**	**57.72**	Hydrophobic	VAL241	A	4.05
		Hydrophobic	VAL241	PA	4.30
		Hydrogen	ARG252	H	1.85, 2.38
		Miscellaneous	MET299	PS	4.99
		Hydrophobic	TYR310	PA	3.99
		Hydrophobic	HIS413	PA	4.18
		Hydrogen	ASP315	H	2.99
		Hydrogen	ARG322	H	1.79
		Hydrophobic	HIS474	PA	5.41
		Hydrophobic	TRP389	PPTs	4.48,
		Unfavorable	MN501	UB	1.48, 2.37
**1b**	11.56	Hydrophobic	VAL241	A	4.64
		Hydrogen	ARG252	H	2.09, 2.52
		Unfavorable	ARG252	UB	1.03, 1.69
		Hydrophobic	MET299	A	4.57
		Miscellaneous	MET299	SX	3.32
		Unfavorable	MET299	UB	1.66
		Hydrophobic	ALA301	A	3.43
		Hydrophobic	TYR310	PPTs	5.63
		Hydrogen	ASP315	H	1.66
		Electrostatic	ARG322	PC	4.08
		Hydrophobic	ILE327	A	4.77
		Hydrophobic	ILE327	PA	4.83
		Hydrophobic	TYR329	PA	4.76
		Hydrophobic	LEU343	A	3.85
		Hydrophobic	VAL376	A	4.61
		Hydrogen	ARG383	H	2.34
		Hydrophobic	ALA385	A	3.60
		Hydrophobic	TRP389	PPTs	5.11
		Unfavorable	MN501	UB	0.36, 1.02, 1.84, 2.10
**OGA**	55.53	Electrostatic	ARG252	AC	3.96
		Hydrogen	TYR329	H	2.10
		Hydrogen	ARG383	H	2.16
		Electrostatic	ARG383	SB	1.67
		Electrostatic	ARG383	AC	1.67
		Miscellaneous	MN501	MA	2.10, 2.21

**1a**: Clitoriacetal; **1b**: Clitoriacetal B; **OGA**: N‐Oxalylglycine (co‐crystallized ligand) MN: manganese(II)ion; AC: Attractive Charge; SB: Salt Bridge; MA: Metal‐Acceptor; A: Alkyl; PA: Pi‐Alkyl; PS: Pi‐Sulfur; PPTs: Pi‐Pi T‐shaped; UB: Unfavorable Bump; SX: Sulfur‐X, PC: Pi‐Cation. GoldScore with rescoring by ChemScore was chosen as a function to evaluate binding efficiency.

Molecular docking simulations indicated a high selectivity of rotenoid 1a for HIF1A, suggesting its potential as a bioactive compound relevant to oncology. This finding not only reinforces the crucial role of computational approaches in drug discovery but also highlights the specific value of molecular docking, which allows for predicting the affinity and specificity of candidate compounds toward biologically relevant therapeutic targets. Similar strategies have been effective in identifying flavonoid derivatives with anticancer activity, as demonstrated by Karoui et al. [52]. Furthermore, theoretical studies by Khettache et al. [53] show that isoflavonoids such as Lupinalbin B exhibit properties like electronic stability, favorable molecular electrostatic potential distribution, and an electron donation tendency. These characteristics are directly associated with the antitumor activity of these compounds. Since rotenoids share a flavonoid‐based structure, they are expected to possess similar characteristics, which would explain their ability to interact with specific cancer‐related proteins like HIF1A. Thus, theoretical findings provide a solid foundation for interpreting the biological activity of rotenoid 1a, highlighting its therapeutic potential in oncological contexts.

## 3 Conclusions

From purifying the ethanolic extract in *Vicia faba* roots, four bioactive compounds were identified—two rotenoids and two isoflavonoids—unrelated to the *Vicia* genus. The rotenoid mixture functioned notably as an antitumor agent against HCT‐116, MCF‐7, and 501Mel cancer cell lines. The mixture did not cause any degree of toxicity toward normal NIH/3T3 cells. Computational target prediction revealed that HIF1A, ESR1, HSP90AB1, and HSP90AA1 are common molecular targets in colorectal cancer, breast cancer, and melanoma and these proteins are associated with tumor progression and with drug resistance. Of the isolated compounds, clitoriacetal showed a strong binding affinity to HIF1A (PDB ID: 6YW3), and this suggests it has a central role in the cytotoxic effects observed via modulation of the HIF1A signaling pathway. These results collectively highlight clitoriacetal as well as suggesting it promises to lead to the development of targeted therapies against multiple cancer types.

## 4 Experimental

### 4.1 General Experimental Procedures

Ultraviolet (UV) data were obtained using an SPD‐M20A Prominence diode array detector module (Shimadzu). IR and VCD experimental spectra were recorded simultaneously with a dual‐PEM Chiral*IR*‐2X FT‐VCD spectrometer (BioTools, Inc.) using a resolution of 4 cm^−1^ and a collection time of 15 h. The optimum retardation of the ZnSe photoelastic modulators (PEMs) was set at 1400 cm^−1^. The IR and VCD spectra of **1a** and **1b** were recorded in CDCl_3_ solution (6 mg in 220 µL) in a BaF_2_ cell with a 100 µm path length. Minor instrumental baseline offsets were eliminated from the final VCD spectra by subtracting the VCD spectrum of the sample from that obtained for the solvent under identical conditions. NMR spectra were obtained using a Bruker Ascend 500 spectrometer (Bruker, Karlsruhe, Germany), with chemical shifts recorded in *δ* (ppm) using the residual peak of the solvent for ^1^H‐NMR or the solvent peak for ^13^C‐NMR as internal standards (CHCl_3_ *δ*_H_ 7.26, central peak of CDCl_3_ *δ*_C_ 77.16). HRESI time‐of‐flight MS (HRESITOFMS) was performed on a Bruker micrOTOF mass spectrometer (Bruker, Karlsruhe, Germany). CC was conducted using Merck silica gel 60 (230−400 mesh, Merck, Darmstadt, Germany) for normal phase separations. Thin‐layer chromatography (TLC) utilized precoated Lemandou silica gel 60 PF254 aluminum back plates (Lemandou, Shijiazhuang, China). Reverse‐phase CC (RP‐CC) was performed with Cosmosil 75C18‐OPN silica gel (Nacalai Tesque Inc., Kyoto, Japan). Fractions obtained from CC were monitored by TLC on aluminum sheets, with spots visualized under UV light at 254 and 366 nm. Commercial‐grade solvents *n*‐hexane, dichloromethane, ethyl acetate, and methanol were distilled before use for extraction and chromatographic purification. HPLC solvents (methanol and acetonitrile) were obtained from Sigma‐Aldrich (Merck, Brazil). The HPLC‐diode array detection system (Shimadzu) was equipped with an SPD‐M20A Prominence Diode Array Detector (Shimadzu) and used a reverse‐phase Kinetex C18 column (5 µm, 250 mm × 4.6 mm, Phenomenex, Torrance, CA, USA) with an FRC‐10A fraction collector.

### 4.2 Plant Material

*V. faba* was harvested in July 2013 at *Mina do Sossego*, *Canaã dos Carajás*, *Pará* state, Brazil (06° 27′ 15.0″ S and 50° 04' 48.2’’ W), and identified by Amanda Cristina D. de Souza from the Laboratory of Systematic Botany (LaBotS) & Herbarium of the Federal University of Rio Grande do Norte, Brazil. The primary tool for taxon identification was the collection available on http://splink.cria.org.br/, with the voucher used for comparison and identification in the system bearing the code INPA 50745. Registration in the National Management System of Genetic Patrimony and Associated Traditional Knowledge (SISGEN) was obtained under the code A4D21AA.

### 4.3 Extraction and Compounds Purification

The plant material was divided into three parts: roots (1874.0 g), leaves (109.0 g), and stems (2394.4 g). Each part underwent drying at 60°C for 48 h followed by grinding. The extractions were carried out using absolute ethanol and left to stand for 72 h. After this period, the solvent was filtered and evaporated in a rotary evaporator. This process was carried out three times, totaling 90.0 g, 70.0 g, and 85.0 g of the roots, leaves, and stems extracts, respectively. The root extract was subjected to liquid‐liquid fractionation with hexane, dichloromethane, and ethyl acetate, resulting in fractions yielding **FHR** (4.0 g), **FDR** (3.9 g), and **FDA** (1.4 g), respectively. The fractionation of **FDR** was performed using classical column chromatography on silica (h = 18 cm, Ø = 5 cm), employing an increasing polarity sequence of mobile phase mixtures comprising hexane, dichloromethane, ethyl acetate, and methanol. This process yielded seven fractions, labeled **FDR** (**1–7**). **FDR‐1** (105.0 mg) was further subjected to column chromatography (h = 22 cm, Ø = 3.5 cm) using hexane, ethyl acetate, and methanol as mobile phases, resulting in ten new fractions, designated as **FDR‐1(*a*–*j*)**. Among these, **FDR‐1*e*** yielded 10.0 mg of compound **2**. The purification of **FDR‐2** (78.5 mg) was also performed using column chromatography with silica (h = 20 cm, Ø = 3.0 cm), employing similar mobile phase compositions consisting of hexane, ethyl acetate, and methanol. This process resulted in nine fractions, labeled **FDR‐2(*a*–*i*)**. Fraction **FDR‐2*b*** (15.1 mg) was purified through semipreparative HPLC (C18 column, Phenomenex Luna, 5 µm, 250 × 10 mm), employing a flow rate of 3.0 mL/min with gradient elution (from 80% to 95% methanol) over 29 min, yielding 2.1 mg of compound **3**. A semipreparative HPLC method was also developed to purify fraction **FDR‐1*f*** (83.6 mg), employing a C18 column (Phenomenex Kinetex, 5 µm, 250 × 10 mm) with a flow rate of 3.3 mL/min and gradient elution ranging from 60% to 95% methanol over 32 min. This process yielded 16.0 mg of the isomers **1a** and **1b**. Several purification strategies were employed to separate the isomers, including chiral chromatography, however, none of those achieved isolated compounds.

Clitoriacetal (**1a**): white amorphous solid; UV (MeOH); λ_max_ (log ε) 293 nm; ^1^H NMR (CDCl_3_, 500 MHz) *δ* 6.65 (1H, s, H‐1), *δ* 6.52 (1H, s, H‐4) *δ* 5.62 (1H, d, J = 2.0 Hz, H‐6), *δ* 4.56 (1H, d, J = 2.0 Hz, H‐6a), *δ* 6.00 (1H, d, J = 2.0 Hz, H‐8), *δ* 6.084 (1H, d, J = 2.0 Hz,, H‐10), *δ* 3.75 (3H, s, 2‐OCH3), *δ* 3.786 (3H, s, 9‐OCH3), *δ* 3.82 (3H, s, 3‐OCH3), *δ* 11.44 (1H, s, 11‐OH); ^13^C NMR (CDCl_3_, 100 MHz) *δ* 108.56 (CH, C‐1), *δ* 144.49 (C, C‐2), *δ* 151.55 (C, C‐3), *δ* 101.16 (CH, C‐4), *δ* 148.11 (C, C‐4a), *δ* 90.4 (CH, C‐6), *δ* 77.06 (CH, C‐6a), *δ* 160.54 (C, C‐7), *δ* 94.81 (CH, C‐8), *δ* 169.32 (C, C‐9), *δ* 95.94 (CH, C‐10), *δ* 164.47 (C, C‐11), *δ* 100.0 (C, C‐11a), *δ* 194.19 (C, C‐12), *δ* 69.71 (C, C‐12a), *δ* 107.71 (C, C‐12b), *δ* 56.38 (CH_3_, 2‐OCH_3_), *δ* 55.99 (CH_3_, 3‐OCH_3_), *δ* 55.93 (CH_3_, 9‐OCH_3_); HREIMS *m/z*  391.1021 (calcd for C_19_H_19_O_9_, 391.1029).

Clitoriacetal b (**1b**): white amorphous solid; UV (MeOH); λ_max_ (log ε) 293 nm; ^1^H NMR (CDCl_3_, 500 MHz) *δ* 6.68 (1H, s, H‐1), *δ* 6.56 (1H, s, H‐4) *δ* 5.73 (1H, d, J = 1.0 Hz, H‐6), *δ* 4.71 (1H, d, J = 1.0 Hz, H‐6a), *δ* 5.97 (1H, d, J = 2.0 Hz, H‐8), *δ* 6.082 (1H, d, J = 2.0 Hz,, H‐10), *δ* 3.76 (3H, s, 2‐OCH_3_), *δ* 3.785 (3H, s, 9‐OCH_3_), *δ* 3.83 (3H, s, 3‐OCH_3_), *δ* 11.39 (1H, s, 11‐OH); ^13^C NMR (CDCl_3_, 100 MHz) *δ* 108.76 (CH, C‐1), *δ* 144.54 (C, C‐2), *δ* 151.91 (C, C‐3), *δ* 102.00 (CH, C‐4), *δ* 148.13 (C, C‐4a), *δ* 91.58 (CH, C‐6), *δ* 74.63 (CH, C‐6a), *δ* 161.014 (C, C‐7), *δ* 94.75 (CH, C‐8), *δ* 169.21 (C, C‐9), *δ* 95.91 (CH, C‐10), *δ* 164.44 (C, C‐11), *δ* 99.85 (C, C‐11a), *δ* 193.15 (C, C‐12), *δ* 68.03 (C, C‐12a), *δ* 107.38 (C, C‐12b), *δ* 56.36 (CH3, 2‐OCH_3_), *δ* 55.97 (CH3, 3‐OCH_3_), *δ* 55.93 (CH_3_, 9‐OCH_3_); HREIMS *m/z*  391.1021 (calcd for C_19_H_19_O_9_, 391.1029).

Alfalone (**2**): yellow amorphous solid; melting point (mp) 242–244°C; UV (MeOH); λ_max_ (log ε) 229, 256, 319 nm; ^1^H NMR (CDCl_3_, 300 MHz) *δ* 6.975 (1H, s, H‐2), *δ* 7.92 (1H, s, H‐5), *δ* 7.65 (1H, s, H‐8), *δ* 7.49 (1H, d, J = 8.7 Hz, H‐2’), *δ* 6.972 (1H, d, J = 8.7 Hz, H‐3’), *δ* 6.972 (1H, d, J = 8.7 Hz, H‐5’), *δ* 7.49 (1H, d, J = 8.7 Hz, H‐6’), *δ* 4.01 (3H, s, 7‐OCH3), *δ* 3.84 (3H, s, 4’‐OCH3); ^13^C NMR (CDCl_3_, 75 MHz) *δ* 152.2 (CH, C‐2), *δ* 124.2 (C, C‐3), *δ* 175.8 (C, C‐4), *δ* 118.0 (C, C‐4a), *δ* 104.9 (CH, C‐5), *δ* 145.5 (C, C‐6), *δ* 152.7 (C, C‐7), *δ* 102.7 (CH, C‐8), *δ* 151.4 (C, C‐8a), *δ* 124.5 (C, C‐1’), *δ* 130.3 (CH, C‐2’), *δ* 114.1 (CH, C‐3’), *δ* 114.1 (CH, C‐5’), *δ* 130.3 (CH, C‐6’), *δ* 56.6 (CH_3_, 7‐OCH_3_), *δ* 55.4 (CH_3_, 4’‐OCH_3_); HREIMS *m/z* 299.0919 (calcd for C_17_H_15_O_5_, 299.0919).

8‐O‐methylretusin (**3**): yellow amorphous solid; mp 231.5–232.5°C; UV (MeOH); λ_max_ (log ε) 215, 252, 316 nm; ^1^H NMR (CDCl_3_, 300 MHz) *δ* 7.97 (1H, s, H‐2), *δ* 7.98 (1H, d, J = 8.7 Hz, H‐5), *δ* 7.04 (1H, d, J = 8.7 Hz, H‐6), *δ* 7.49 (1H, d, J = 9.0 Hz, H‐2’), *δ* 6.97 (1H, d, J = 9.0 Hz, H‐3’), *δ* 6.97 (1H, d, J = 9.0 Hz, H‐5’), *δ* 4.08 (3H, s, 8‐OCH3), *δ* 3.84 (3H, s, 4’‐OCH3); ^13^C NMR (DMSO‐d6, 100 MHz) *δ* 153.0 (CH, C‐2), *δ* 122.9 (C, C‐3), *δ* 174.7 (C, C‐4), *δ* 117.4 (C, C‐4a), *δ* 120.7 (CH, C‐5), *δ* 115.2 (C, C‐6), *δ* 154.7 (C, C‐7), *δ* 134.7 (CH, C‐8), *δ* 150.7 (C, C‐8a), *δ* 124.1 (C, C‐1’), *δ* 130.1 (CH, C‐2’), *δ* 113.6 (CH, C‐3’), *δ* 113.6 (CH, C‐5’), *δ* 130.1 (CH, C‐6’), *δ* 60.7 (CH_3_, 8‐OCH_3_), *δ* 55.1 (CH_3_, 4’‐OCH_3_); HREIMS *m/z* 299.0916 (calcd for C_17_H_15_O_5_, 299.0919).

### 4.4 VCD Calculations

The conformational searches 1a and 1b were carried out at the molecular mechanics level of theory employing the Merck molecular force field (MMFF) incorporated Spartan 08 software package. The DFT calculations were carried out at 298 K in chloroform solution using the polarizable continuum model (PCM) in its integral equation formalism version (IEFPCM), incorporated in Gaussian 09 software (Revision A.02). The (6R,6aS,12aR) and (6S,6aS,12aR) configurations were arbitrarily chosen for 1a and 1b, respectively. Initially, 45 and 100 conformers were identified within a 10 kcal/mol energy window for **1a** and **1b**, respectively, and geometry optimized at the B3PW91/PCM(CHCl3)/6‐311G(d,p) level. The 6 lowest‐energy conformers of each compound with relative energy (rel E.) < 1.9 kcal/mol (Figures S12 and S13), were then selected for IR/VCD spectral simulations, which were calculated at the same level used in the geometry optimization steps. IR and VCD spectra were created using dipole and rotational strengths from Gaussian and converted into molar absorptivity (M^−1^ cm^−1^). Each spectrum was plotted as a sum of Lorentzian bands with half‐widths at half‐maximum (HWHM) of 6 cm^−1^. The calculated wavenumbers were multiplied with a scaling factor of 0.98 and the simple average spectra were plotted using Origin software.

### 4.5 Cytotoxicity Assay

Compounds were assessed for cytotoxicity against human tumor cell lines HCT‐116 (colorectal adenocarcinoma; ATCC CCL 247), 501Mel (melanoma; RRID: CVCL_4633), and MCF‐7 (breast adenocarcinoma; ATCC HTB 22), along with non‐tumor cells line NIH/3T3 (mouse embryonic fibroblast; ATCC CRL 1658). Cells were maintained in optimal media (RPMI 1640 for HCT‐116 and 501Mel; DMEM for NHI/3T3; and DMEM‐F12 for MCF‐7), supplemented with 10% fetal bovine serum (FBS) and 1% antibiotics (penicillin and streptomycin). Cells were kept at 37°C in a humidified atmosphere containing 5% CO_2_. For the MTT assay, cells were seeded into 96‐well plates at a density of 6 × 10^3^ or 2 × 10^3^ cells/well for HCT‐116. After 24 h, the mixture **1a** and **1b**, compounds **2** and **3**, at concentrations ranging from 0.0032 to 50 µM, were added to each well in duplicates and incubated for 72 h. Dimethyl sulfoxide (DMSO) (0.05%) served as the negative control, while the chemotherapeutic agent doxorubicin (0.00064–10 µM) was the positive control. At the end of the incubation period, the culture medium was replaced with a fresh medium containing MTT solution (0.5 mg/mL) and further incubated for 3 h. The MTT solution was then removed, and the formazan crystals formed were dissolved in 150 µL of DMSO. Absorbance for each well was measured at 570 nm, and then the values thereof were normalized in the percentage of viable cells based on that of the negative control. IC_50_ values and their respective 95% CIs and R^2^ were calculated using sigmoidal nonlinear regression with GraphPad Prism 8.0 software considering the results of three different experiments. SIs were obtained as the ratio of the IC_50_ value for the respective tumor cell line to that of the NIH/3T3 cell line.

### 4.6 Identification of Potential Targets of Rotenoids

The 2D structures of the compounds were generated using MarvinSketch software (version 24.3.0) and exported as a single SMILES file. The SMILES representation of each **1a** and **1b** structure was utilized for target prediction via the Swiss Target Prediction database (http://www.swisstargetprediction.ch/), considering Homo sapiens as the species and a selection threshold of probability >0.1. Additional predictions were performed using Similarity Ensemble Approach (SEA) (https://sea.bkslab.org/), Way2Drug PASS Online (http://www.way2drug.com/passonline), TargetNet (http://targetnet.scbdd.com/calcnet/index/), and SuperPred (https://prediction.charite.de/index.php). Duplicate genes were removed, and all identified targets were consolidated. The UniProt database (https://www.uniprot.org/) was used to convert gene names into protein names.

### 4.7 Screening for Targets in Colorectal Cancer, Breast Cancer, and Melanoma

To identify targets associated with colorectal cancer, breast cancer, and melanoma, we conducted comprehensive searches using Online Mendelian Inheritance in Man (OMIM, https://omim.org/), GeneCards (https://www.genecards.org/), DrugBank (https://go.drugbank.com/), and the Therapeutic Target Database (TTD, http://db.idrblab.net/ttd/). By intersecting the targets of the rotenoid mixture with those associated with these diseases using Venny 2.1 (http://www.bioinformatics.com.cn/static/others/jvenn/example.html), we identified potentially relevant targets for treating these cancers. The results were imported into Cytoscape software (version 3.10.3) for visualization.

### 4.8 Construction PPI Network

The previously identified common targets were uploaded to the STRING online database (https://string‐db.org/), selecting “Homo sapiens” as the species and setting a medium confidence score threshold of 0.7 to construct a PPI network. The resulting PPI network was then exported as a “tsv” file and imported into Cytoscape 3.10.3, where the top 20 Hub genes with high connectivity in the PPI network were screened out by the MCC method using Cytohubba plug‐in of Cytoscape software.

### 4.9 GO and KEGG Pathway Enrichment Analyses

GO and KEGG pathway analyses were conducted using the Database for Annotation, Visualization, and Integrated Discovery (DAVID), a web‐based gene set enrichment analysis platform (https://david.ncifcrf.gov/). GO analysis included three main categories: BP, CC, and MF, along with KEGG enrichment analysis. The results were visualized using a free online platform for GO and KEGG enrichment analysis visualization (http://www.bioinformatics.com.cn), where the top 10 GO terms and the top 20 KEGG pathways were plotted. *p*‐Value was calculated in GO and KEGG analyses, and *p* < 0.05 suggests the enrichment degree was statistically significant and the pathway results would certainly be necessary functional mechanisms of cancer.

### 4.10 Molecular Docking

The mixture, composed of clitoriacetal (**1a**) and clitoriacetal B (**1b**), was designed in MarvinSketch (Chemaxon, v.24.3.0) with pH adjustments based on the physiological pH of each protein target (PDB) and optimized in Avogadro 1.2.0 (MMFF94 force field, dE = 1 × 10⁻⁷ kJ/mol) [54], then exported as MOL2 files. Protein structures were obtained from the Research Collaboratory for Structural Bioinformatics Protein Data Bank (RCSB PDB) (http://www.rcsb.org/pdb), and surface charge distributions at physiological pH were calculated by employing the adaptive Poisson–Boltzmann solver and PDB2PQR servers (https://server.poissonboltzmann.org/pdb2pqr), with the PARSE force field [55]. Molecular docking simulations were performed using GOLD v.2024.1.0. The scoring strategy employed GoldScore as the primary scoring function, complemented by ChemScore for rescoring, in a consensus scoring approach. GoldScore primarily evaluates van der Waals interactions and hydrogen bonding, while ChemScore incorporates estimates of binding energy, including contributions from hydrogen bonding, metal‐ion interactions, lipophilic interactions, and the loss of conformational entropy, alongside the geometric complementarity between the ligand and receptor [56]. This combination has been previously demonstrated to improve docking accuracy and ranking reliability [57, 58]. Given that both scoring functions generate values in arbitrary units, the absolute docking scores cannot be directly compared with those from other docking programs. Instead, these scores serve as relative indicators of binding affinity within the same scoring framework. Each docking experiment consisted of 10 independent runs per structure, with poses ranked based on scoring functions and prevalence, analyzed in Discovery Studio Visualizer v24.1.0.23298 (BIOVIA). Docking predictions with a root mean square deviation of less than 2.0 Å were considered successful. All water molecules were removed before docking, and the active site was defined by the geometric center of the co‐crystallized ligand within a 15 Å spherical grid. The results were analyzed and visualized using Discovery Studio Visualizer v24.1.0.23298 and UCSF Chimera v1.18.

## Author Contributions

**Victor Menezes Sipoloni**: data curation, writing – original draft, review & editing. **João Victor Silva‐Silva**: data curation, writing – original draft, review & editing. **Elthon G. Ferreira**: data curation. **Eric Yoshitaka Lee**: data curation. **João Marcos Batista Junior**: data curation, writing – original draft, review & editing. **Miriam Uemi**: data curation. **Lívia Soman de Medeiros**: supervision, writing – original draft, review & editing. **Paula C. Jimenez**: data curation, writing – review & editing, supervision, resources. **Thiago A. M. Veiga**: writing – review & editing, supervision, resources.

## Conflicts of Interest

The authors declare no conflicts of interest.

## Supporting information



Supporting Information for this article is available on the WWW under https://doi.org/10.1002/MS‐number.

## Data Availability

The authors have nothing to report.
