# Supplementary material for: Rotenoids from the Roots of Vicia faba L. (Fabaceae): Structural Characterization, Cytotoxic Effects, and Molecular Docking
Source: Chem Biodivers. 2025 May 26;22(10):e01091. doi: 10.1002/cbdv.202501091 (PMC12533803; doi:10.1002/cbdv.202501091)
Supplement: Supplementary file 1 — Supporting Information for this article is available on the WWW under https://doi.org/10.1002/MS‐number. [file CBDV-22-e01091-s001.pdf]

Supporting Information

List of Contents

Figure S1. Mass spectrum (MS^2) of the m/z 391.1021 ion, obtained in positive ion mode using ESI 2
Figure S2. ^1H NMR spectrum ($CDCl_3$, 500 MHz) of **1a** and **1b**. 2
Figure S3. HMBC contour map ($CDCl_3$, 500 MHz) for **1a** and **1b**........ 3
Figure S4. HSQC contour map ($CDCl_3$, 500 MHz) for **1a** and **1b**........ 4
Figure S5. COSY (^1H – ^1H) ($CDCl_3$, 500 MHz) for **1a** and **1b** 4
Figure S6. NOESY contour map ($CDCl_3$, 500 MHz) for **1a** and **1b**........ 5
Figure S7. Long-distance correlations observed through the 2D NOESY experiment for **1a** and **1b**........ 6
Figure S8. Comparison between experimental IR and VCD spectra for the mixture **1a**+**1b** (black trace) with calculated B3PW91/PCM(CHCl3)/6-311G(d,p) data for individual (6R,6aS,12aR)-**1a**.7
Figure S9. Comparison between experimental IR and VCD spectra for the mixture **1a**+**1b** (black trace) with calculated B3PW91/PCM(CHCl3)/6-311G(d,p) data for individual (6S,6aS,12aR)-**1b**.8
Figure S10. Venn diagram of potential targets for cancer treatment using mixture of **1a** and **1b**. 9
Figure S11. PPI network for cancer treatment using the mixture of **1a** and **1b**. (A) PPI network analysis. (B) Top 20 nodes are the likely key proteins in the interaction. 10
Figure S12. Optimized structures, relative energies (DG_{298K}), and Boltzmann populations (%) of the lowest-energy conformers identified for (6R,6aS,12aR)-**1a** at the B3PW91/PCM(CHCl3)/6-311G(d,p) level. Referenced to G = -885647.1515 kcal/mol........ 11
Figure S13. Optimized structures, relative energies (DG_{298K}), and Boltzmann populations (%) of the lowest-energy conformers identified for (6S,6aS,12aR)-**1b** at the B3PW91/PCM(CHCl3)/6-311G(d,p) level. Referenced to G = -885648.0501 kcal/mol........ 11

Table S1. Functions of potential target genes based on KEGG pathway analysis. 12

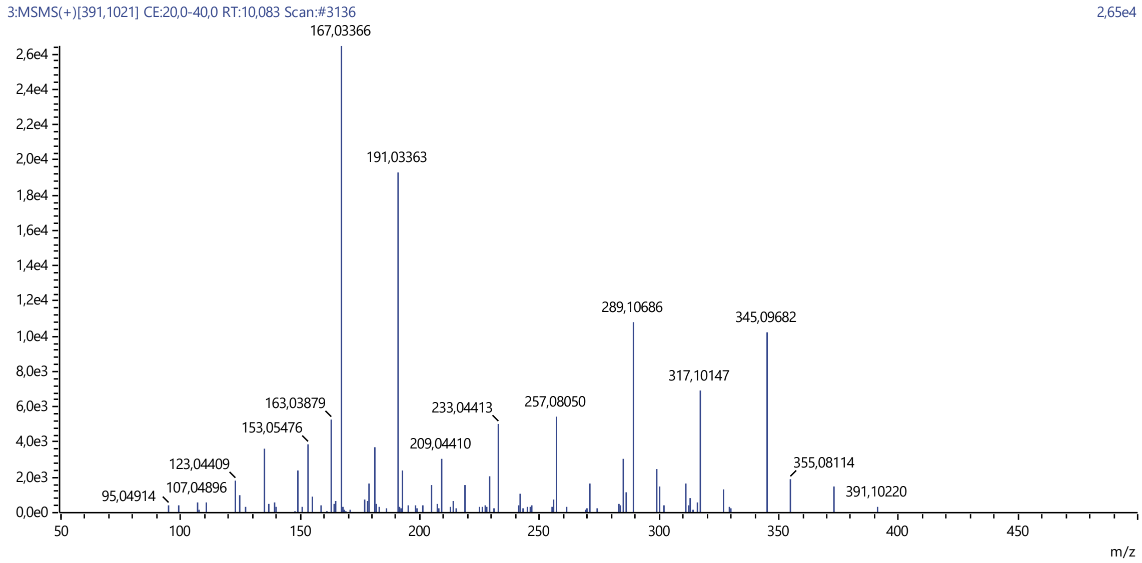

Figure S1. Mass spectrum (MS^2) of the *m/z* 391.1021 ion, obtained in positive ion mode using ESI

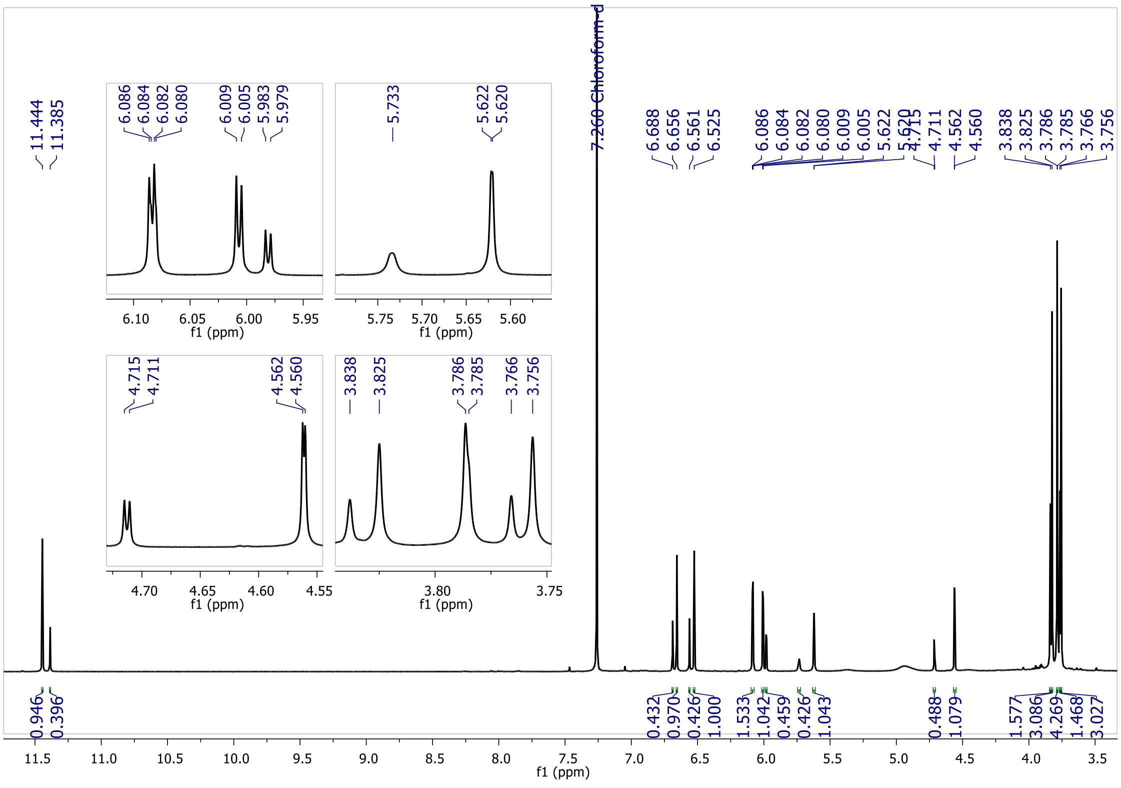

Figure S2. ^{1}H NMR spectrum ($CDCl_3$, 500 MHz) of **1a** and **1b**.

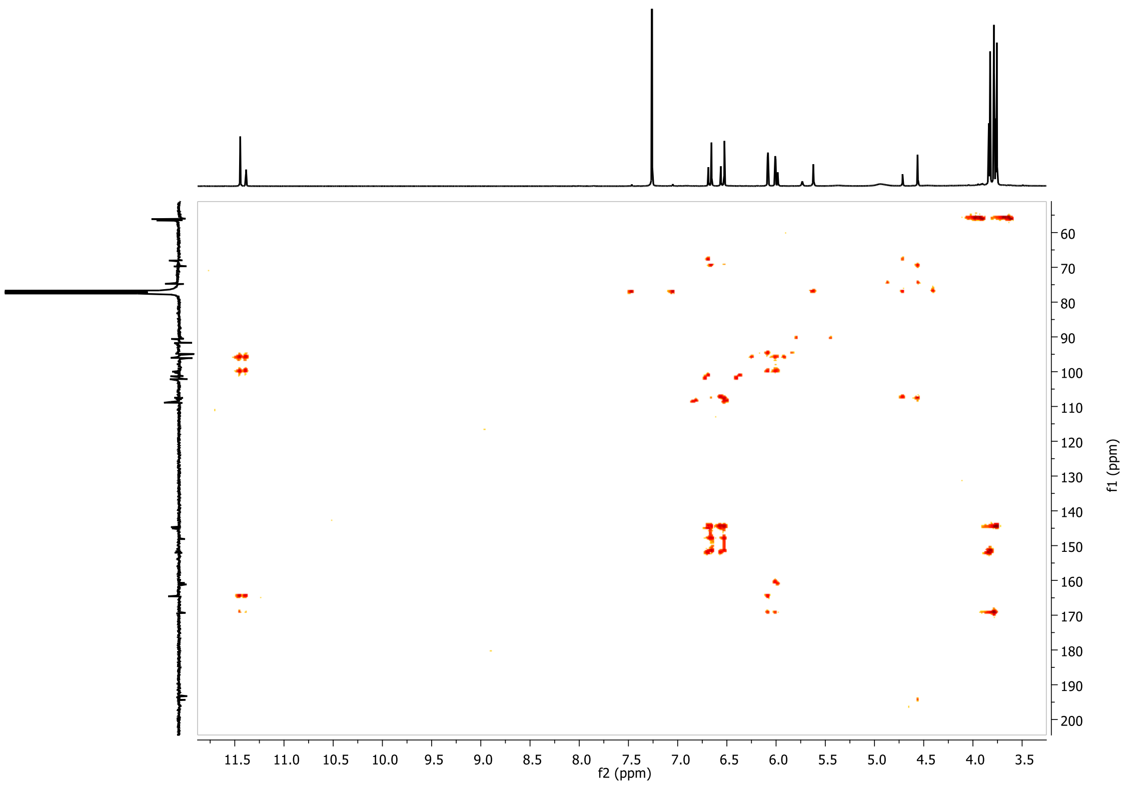

Figure S3. HMBC contour map ($CDCl_3$, 500 MHz) for **1a** and **1b**

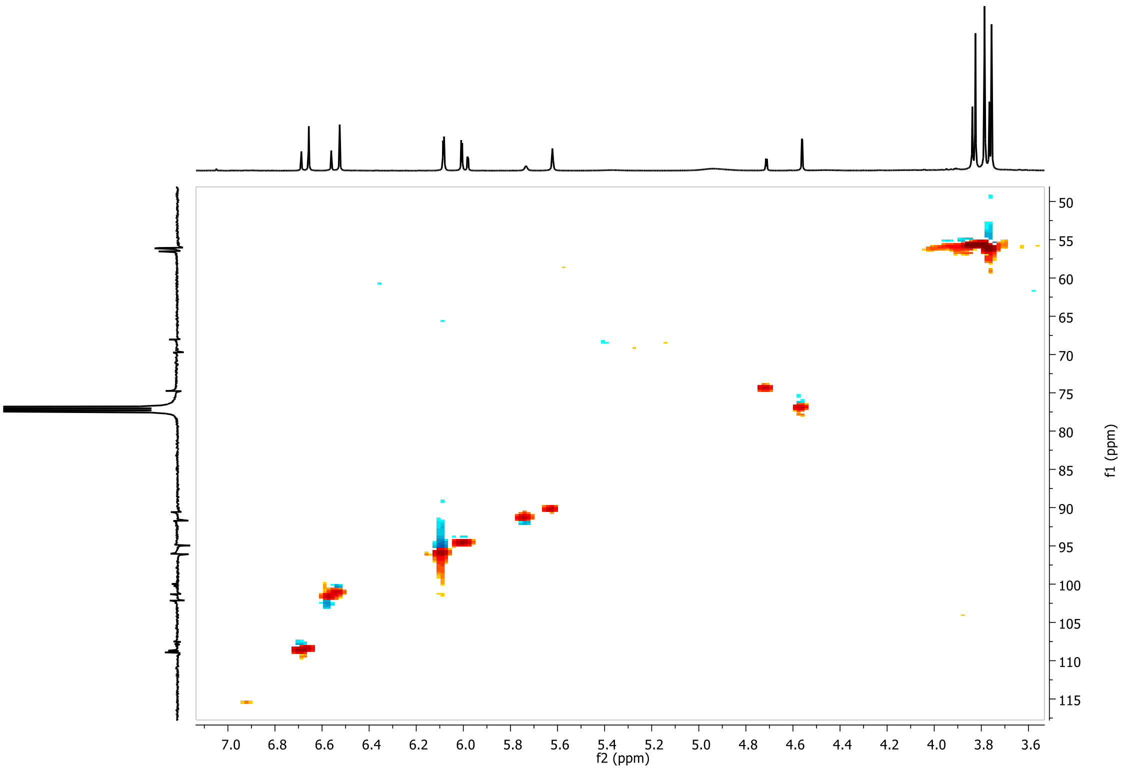

Figure S4. HSQC contour map ($CDCl_3$, 500 MHz) for **1a** and **1b**.

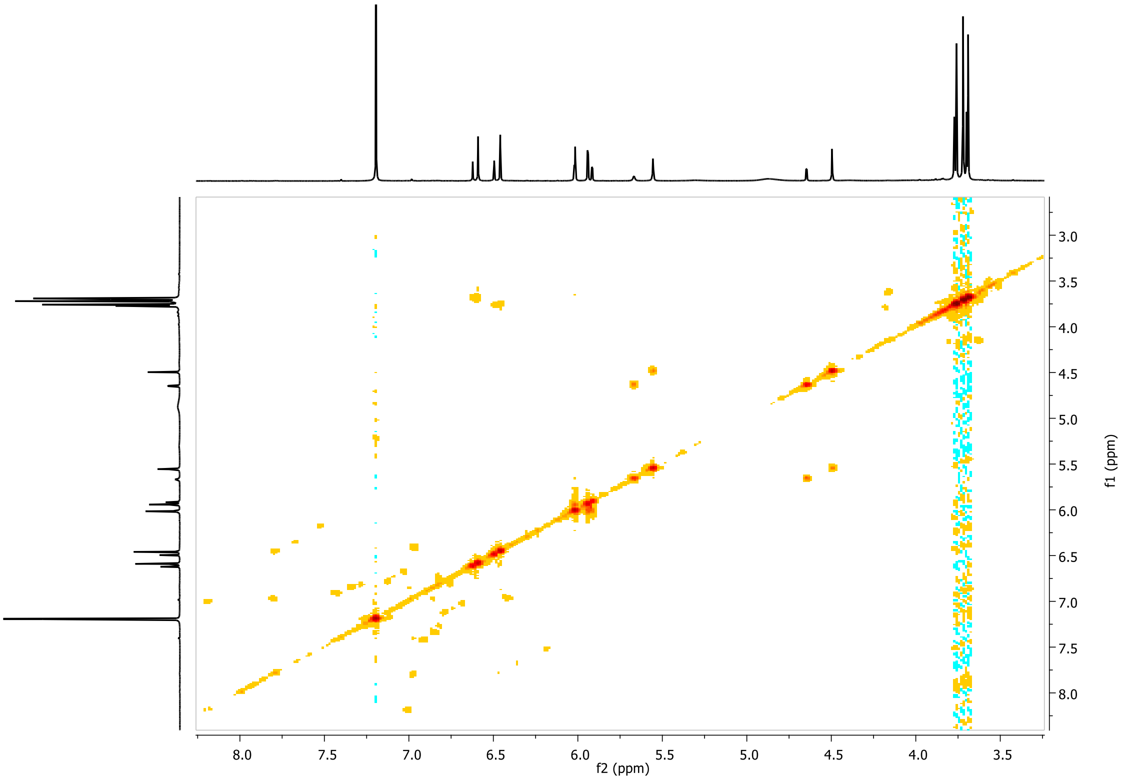

Figure S5. COSY ($^{1}H - ^{1}H$) ($CDCl_3$, 500 MHz) for **1a** and **1b**

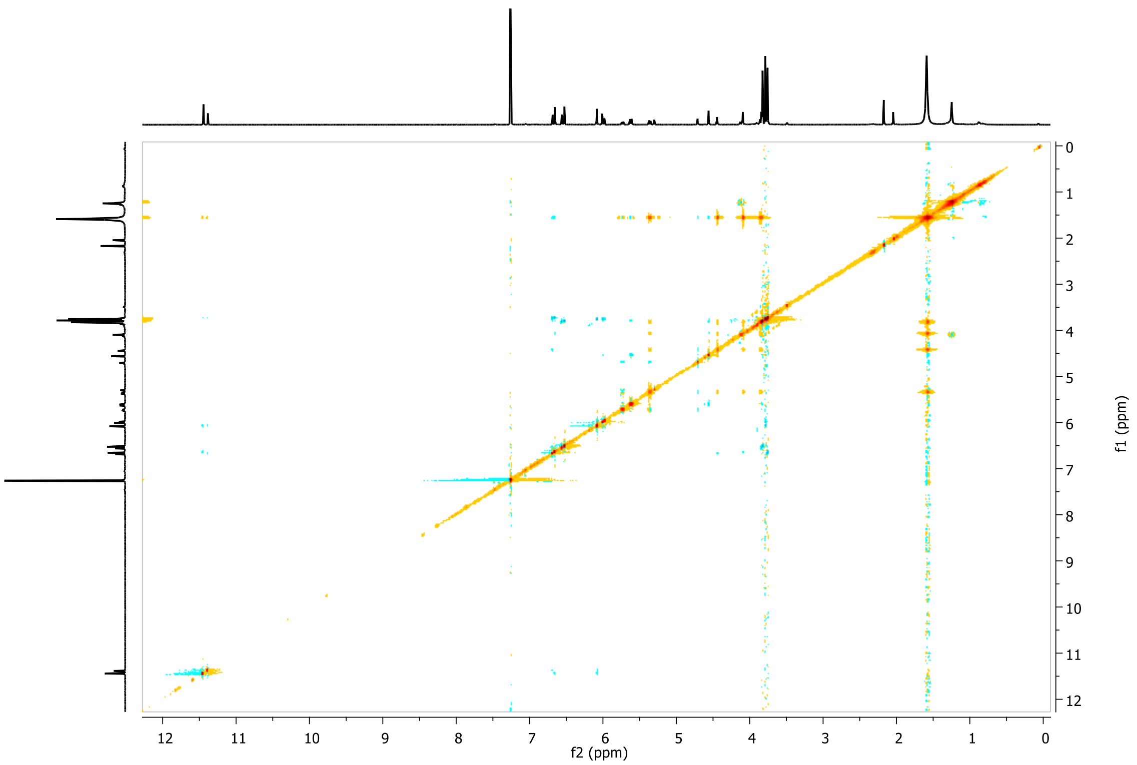

Figure S6. NOESY contour map ($CDCl_3$, 500 MHz) for **1a** and **1b**

1a

1b

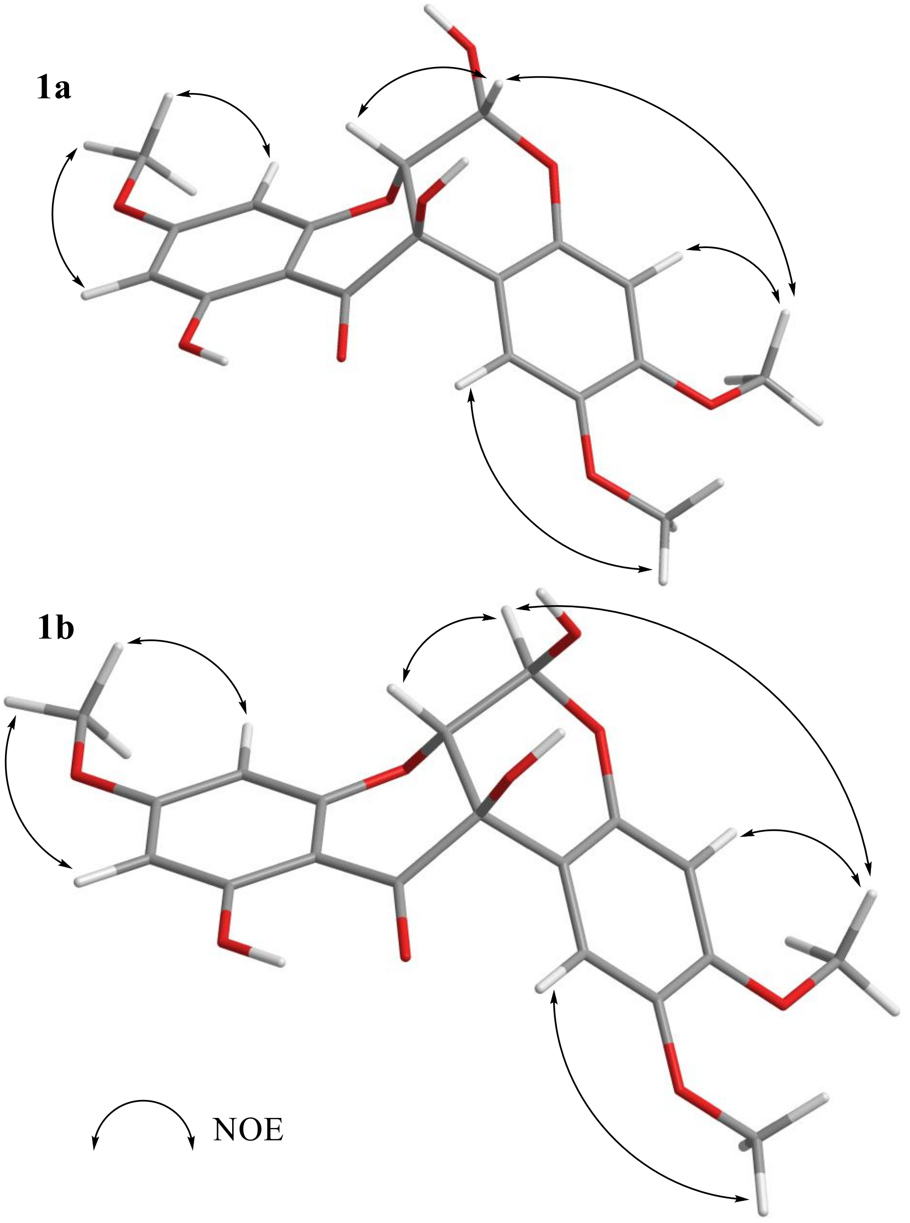

Figure S7. Long-distance correlations observed through the 2D NOESY experiment for **1a** and **1b**.

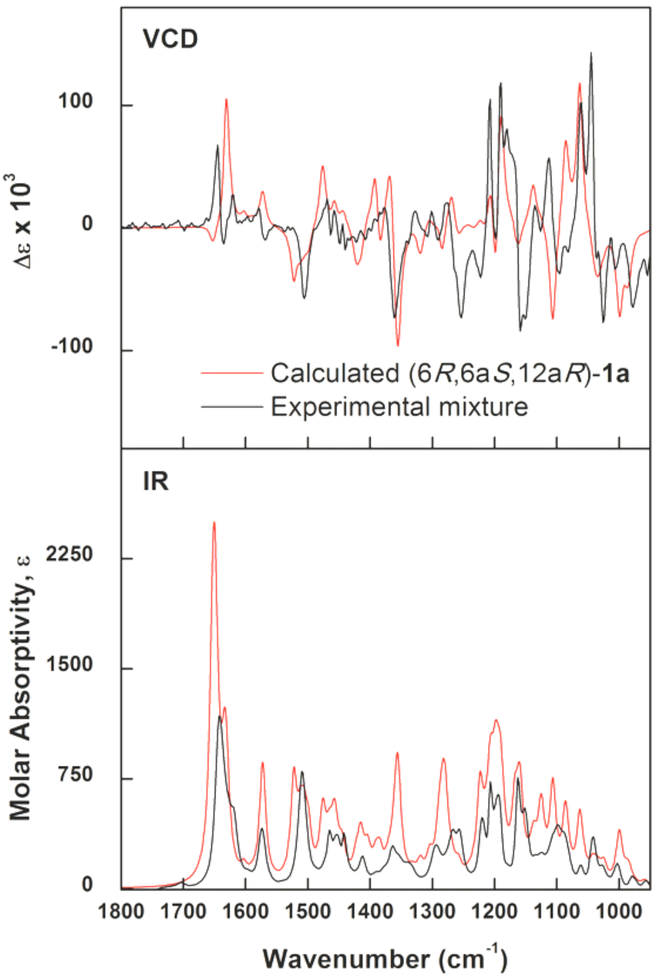

Figure S8. Comparison between experimental IR and VCD spectra for the mixture **1a**+**1b** (black trace) with calculated B3PW91/PCM(CHCl3)/6-311G(d,p) data for individual (6R,6aS,12aR)-**1a**.

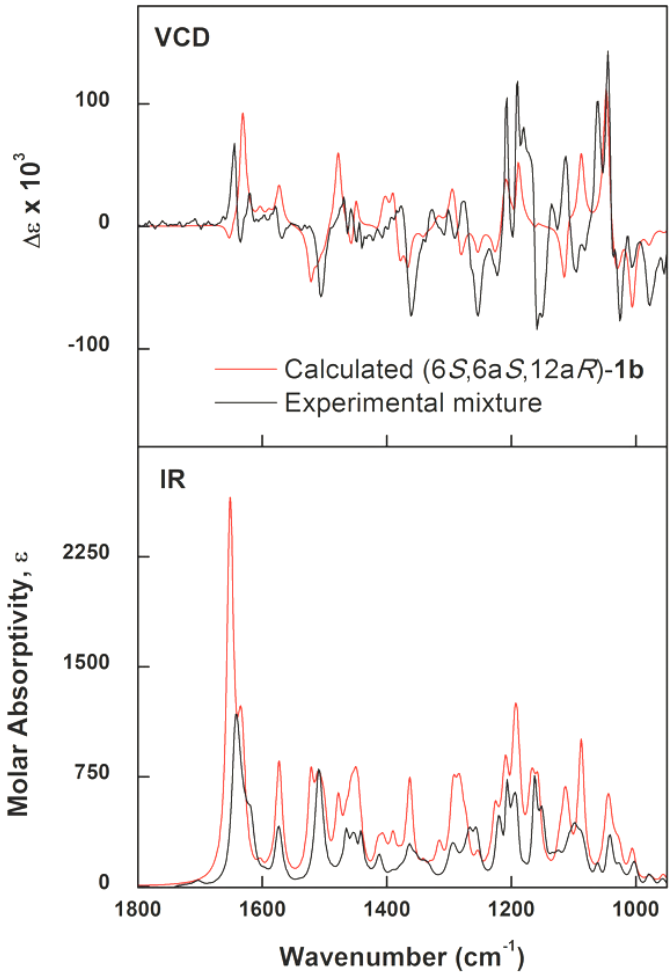

Figure S9. Comparison between experimental IR and VCD spectra for the mixture **1a**+**1b** (black trace) with calculated B3PW91/PCM(CHCl3)/6-311G(d,p) data for individual (6S,6aS,12aR)-**1b**.

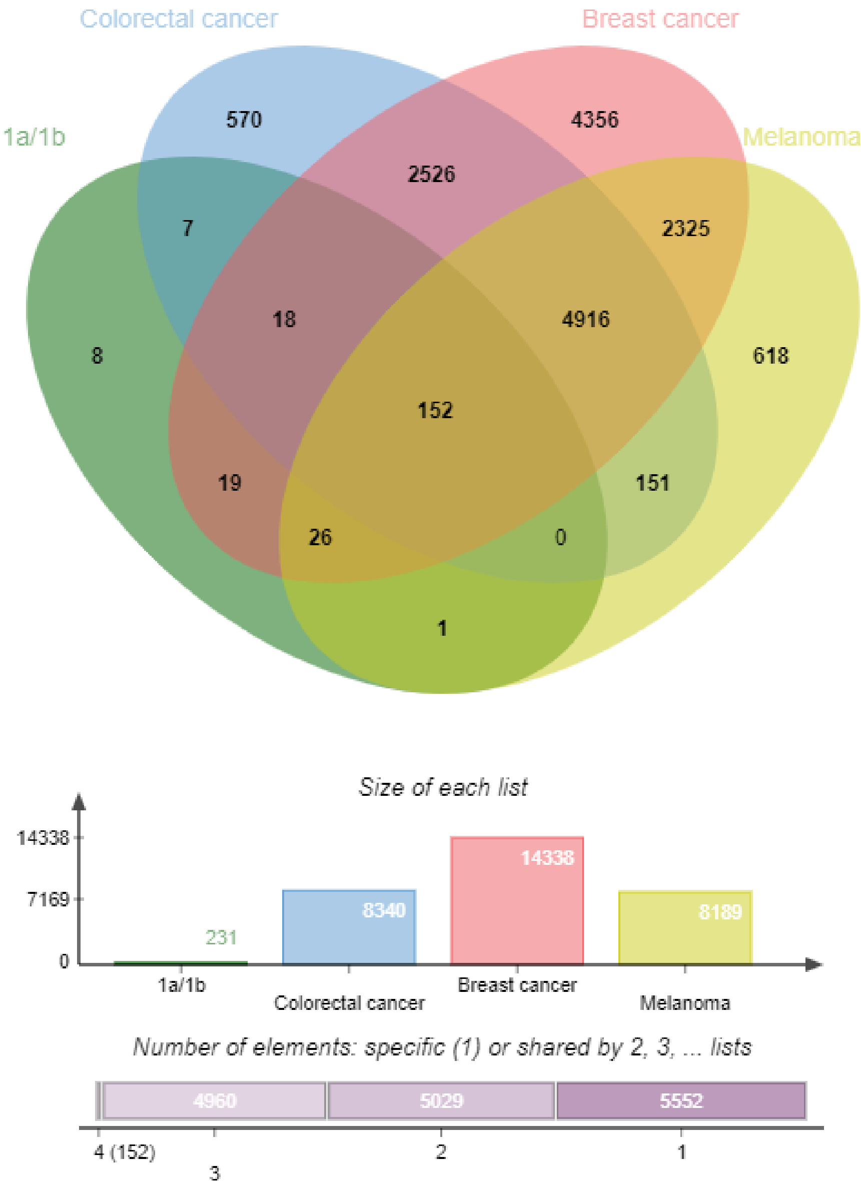

Figure S10. Venn diagram of potential targets for cancer treatment using mixture of **1a** and **1b**.

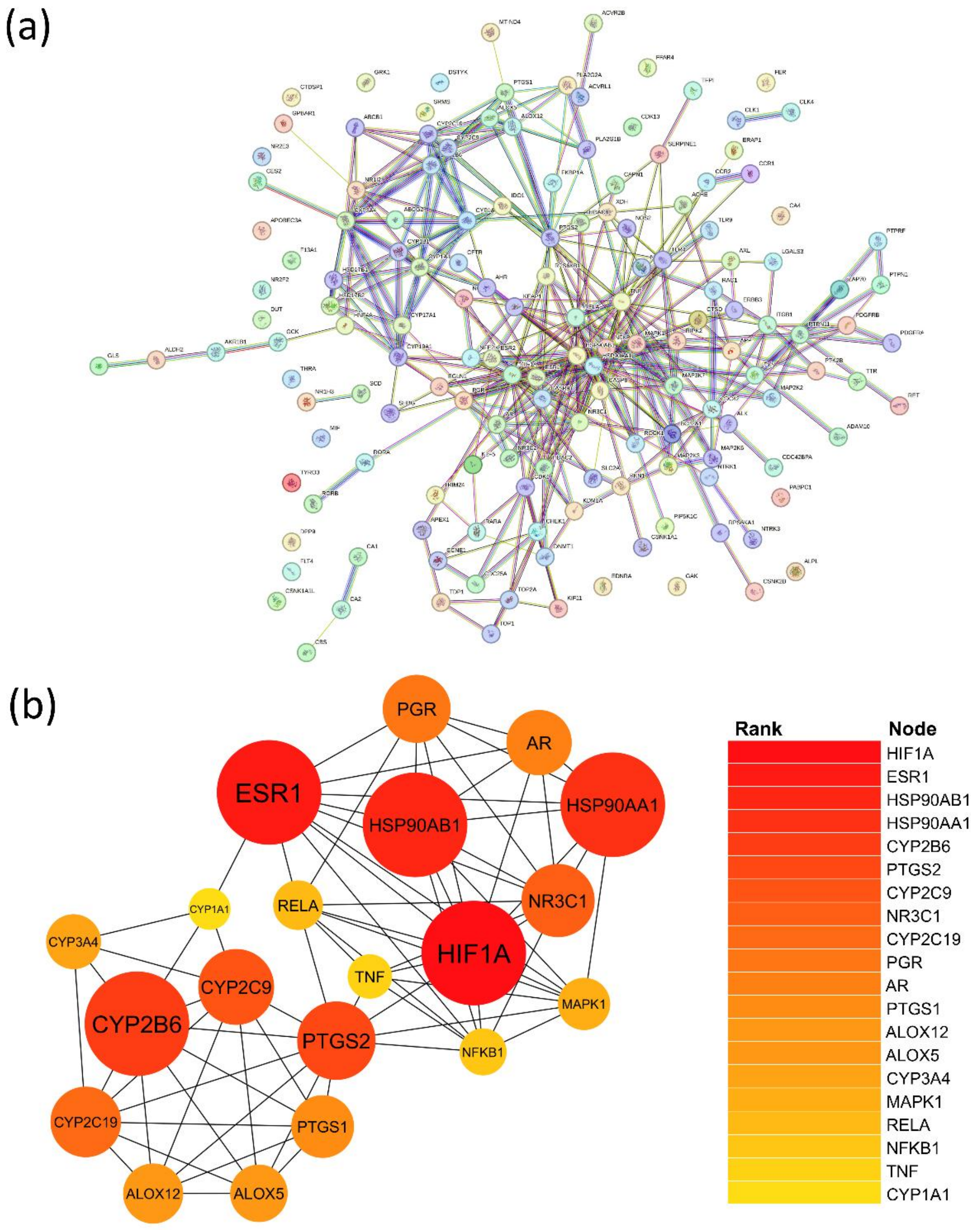

Figure S11. PPI network for cancer treatment using the mixture of **1a** and **1b**. (A) PPI network analysis. (B) Top 20 nodes are the likely key proteins in the interaction.

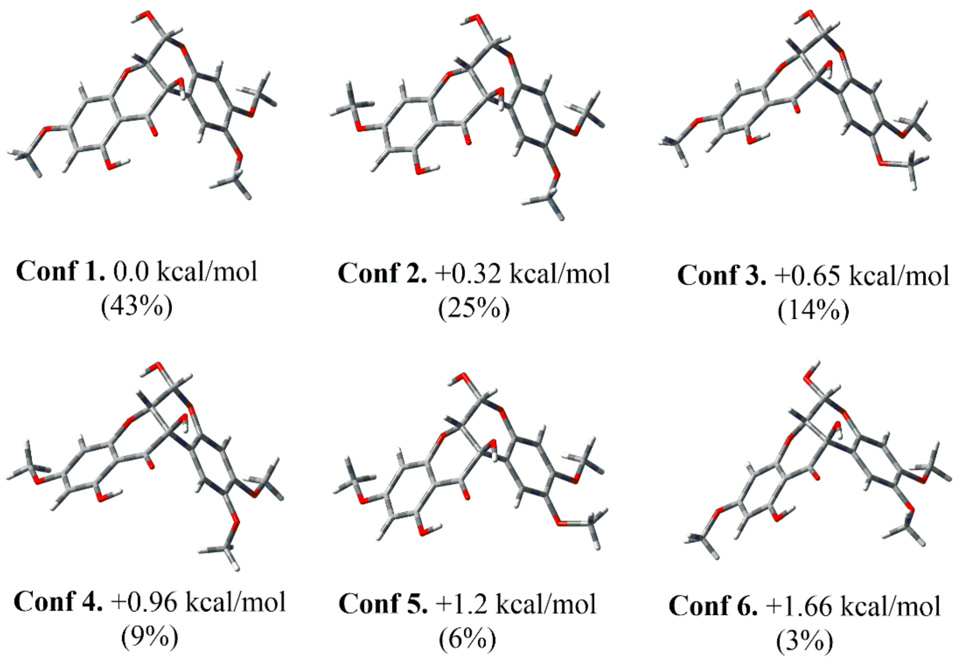

Figure S12. Optimized structures, relative energies (DG_{298K}), and Boltzmann populations (%) of the lowest-energy conformers identified for (6R,6aS,12aR)-**1a** at the B3PW91/PCM(CHCl3)/6-311G(d,p) level. Referenced to G = -885647.1515 kcal/mol.

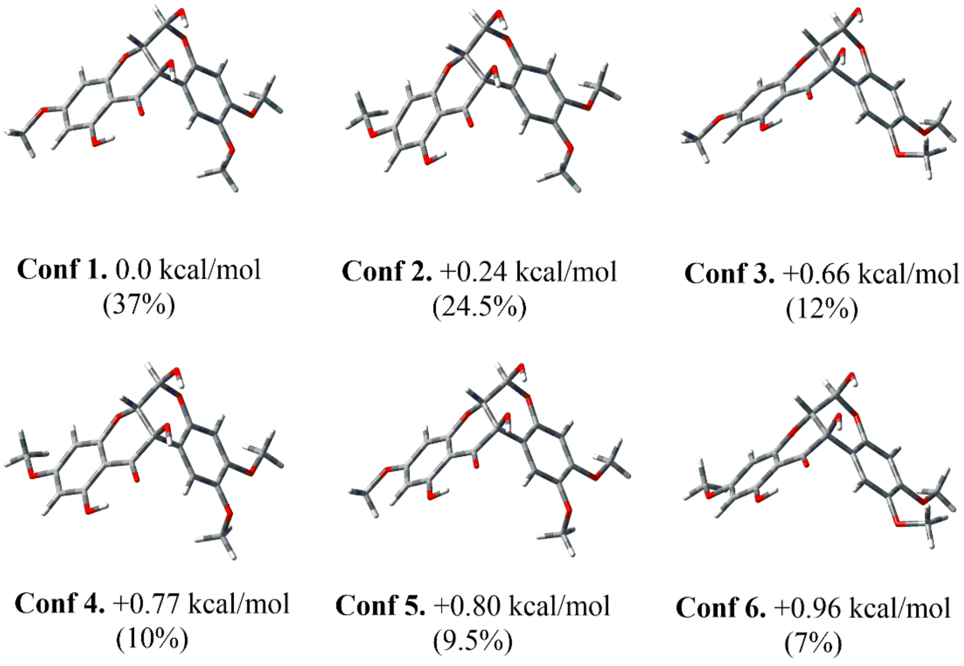

Figure S13. Optimized structures, relative energies (DG_{298K}), and Boltzmann populations (%) of the lowest-energy conformers identified for (6S,6aS,12aR)-**1b** at the B3PW91/PCM(CHCl3)/6-311G(d,p) level. Referenced to G = -885648.0501 kcal/mol.

Table S1. Functions of potential target genes based on KEGG pathway analysis.

Term	Number of pathway gene	*P*-Value
Pathways in cancer	RET, ITGB1, ALK, HDAC2, HSP90AB1, ROCK1, FLT4, SLC2A1, KEAP1, PTGS2, HIF**1a**, RELA, CASP9, CDC42, EDNRA, CASP8, MAPK1, RAC1, PDGFRB, NTRK1, EGLN1, NQO1, PDGFRA, HSP90AA1, MAP2K2, NOS2, ESR1, ESR2, NFKB1, AR, RPS6KB1, CCNE1, RARA, NFE2L2	1.25E-12
Yersinia infection	MAP2K3, ITGB1, MAP2K2, ROCK1, TNF, RELA, NFKB1, CDC42, ZAP70, RPS6KA1, PTK2B, MAPK1, PIP5K1C, PKN1, RAC1, MAP3K7, TLR4, MAP2K6	1.83E-11
Chemical carcinogenesis - receptor activation	HSP90AA1, MAP2K2, HSP90AB1, AHR, CYP3A4, ESR1, CDC25A, RELA, ESR2, NFKB1, AR, KLF5, CYP2B6, RPS6KB1, CYP**1a**2, RPS6KA1, CYP**1a**1, CYP**1b**1, MAPK1, PGR	3.88E-10
PD-L1 expression and PD-1 checkpoint pathway in cancer	MAP2K3, ALK, MAP2K2, PTPN11, HIF**1a**, RELA, NFKB1, ZAP70, RPS6KB1, CSNK2B, TLR9, MAPK1, TLR4, MAP2K6	6.65E-10
Lipid and atherosclerosis	MAP2K3, HSP90AA1, HSP90AB1, TNF, RELA, NFKB1, CDC42, CASP9, CYP2C9, CASP8, CYP2B6, CYP**1a**1, MAPK1, RAC1, MAP3K7, TLR4, NOX1, MAP2K6, NFE2L2	3.02E-09
Central carbon metabolism in cancer	NTRK1, PDGFRB, RET, PDGFRA, LDHB, MAP2K2, NTRK3, SLC2A1, MAPK1, HIF**1a**, GCK, GLS	6.81E-09
Toxoplasmosis	MAP2K3, ITGB1, NOS2, TNF, RELA, NFKB1, CASP9, CASP8, ALOX5, MAPK1, MAP3K7, TLR4, MAP2K6	9.74E-08
Human immunodeficiency virus 1 infection	MAP2K3, MAP2K2, TNF, RELA, NFKB1, CASP9, CASP8, RPS6KB1, CHEK1, CDK1, PTK2B, MAPK1, RAC1, MAP3K7, TLR4, APOBEC3A, MAP2K6	1.05E-07
Chemical carcinogenesis - reactive oxygen species	NQO1, PTPN1, MT-ND4, MAP2K2, KEAP1, PTPN11, AHR, HIF**1a**, RELA, NFKB1, CYP**1a**2, CYP**1a**1, CYP**1b**1, MAPK1, RAC1, NOX1, NFE2L2	2.53E-07
Toll-like receptor signaling pathway	MAP2K3, MAP2K2, CASP8, TLR9, MAPK1, RAC1, TNF, MAP3K7, TLR4, RELA, NFKB1, MAP2K6	6.29E-07
HIF-1 signaling pathway	EGLN1, LDHB, MAP2K2, RPS6KB1, NOS2, SERPINE1, SLC2A1, MAPK1, HIF**1a**, TLR4, RELA, NFKB1	6.29E-07
Salmonella infection	MAP2K3, HSP90AA1, MAP2K2, HSP90AB1, RIPK2, TNF, RELA, NFKB1, CDC42, CASP8, TLR9, MAPK1, PKN1, RAC1, MAP3K7, TLR4, MAP2K6	9.82E-07
Fluid shear stress and atherosclerosis	NQO1, HSP90AA1, HSP90AB1, KEAP1, TNF, ACVR2B, RELA, NFKB1, RAC1, MAP3K7, NOX1, MAP2K6, NFE2L2	1.21E-06
Prostate cancer	PDGFRB, CASP9, PDGFRA, AR, HSP90AA1, MAP2K2, HSP90AB1, CCNE1, MAPK1, RELA, NFKB1	1.88E-06
Arachidonic acid metabolism	CYP2C9, CYP2B6, PLA2G**1b**, ALOX5, PLA2G2A, ALOX12, CYP2C19, PTGS2, PTGS1	4.02E-06
Hepatitis B	MAP2K3, MAP2K2, TNF, RELA, NFKB1, CASP9, CASP8, CCNE1, PTK2B, MAPK1, MAP3K7, TLR4, MAP2K6	5.59E-06
Human cytomegalovirus infection	CCR1, PDGFRA, MAP2K2, ROCK1, PTGS2, TNF, RELA, NFKB1, CASP9, CASP8, RPS6KB1, PTK2B, MAPK1, RAC1, MAP2K6	6.82E-06
PI3K-Akt signaling pathway	PDGFRB, ITGB1, NTRK1, RET, PDGFRA, HSP90AA1, MAP2K2, HSP90AB1, FLT4, RELA, NFKB1, CASP9, ERBB3, RPS6KB1, CCNE1, MAPK1, PKN1, RAC1, TLR4	7.03E-06
Adherens junction	CDC42, PTPN1, FER, ROCK1, CSNK2B, MAPK1, FYN, RAC1, MAP3K7, PTPRF	9.73E-06
MAPK signaling pathway	MAP2K3, PDGFRB, NTRK1, RET, PDGFRA, MAP2K2, FLT4, TNF, RELA, NFKB1, CDC42, ERBB3, RPS6KA1, MAPK1, RAC1, MAP3K7, MAP2K6	9.92E-06
